# A lipid-associated macrophage lineage rewires the spatial landscape of adipose tissue in early obesity

**DOI:** 10.1172/jci.insight.171701

**Published:** 2023-10-09

**Authors:** Cooper M. Stansbury, Gabrielle A. Dotson, Harrison Pugh, Alnawaz Rehemtulla, Indika Rajapakse, Lindsey A. Muir

**Affiliations:** 1 Department of Computational Medicine and Bioinformatics,; 2 The Michigan Institute for Computational Discovery and Engineering,; 3 Department of Radiation Oncology, and; 4 Department of Mathematics, University of Michigan, Ann Arbor, Michigan, USA.

**Keywords:** Metabolism, Adipose tissue, Macrophages, Obesity

## Abstract

Adipose tissue macrophage (ATM) infiltration is associated with adipose tissue dysfunction and insulin resistance in mice and humans. Recent single-cell data highlight increased ATM heterogeneity in obesity but do not provide a spatial context for ATM phenotype dynamics. We integrated single-cell RNA-Seq, spatial transcriptomics, and imaging of murine adipose tissue in a time course study of diet-induced obesity. Overall, proinflammatory immune cells were predominant in early obesity, whereas nonresident antiinflammatory ATMs predominated in chronic obesity. A subset of these antiinflammatory ATMs were transcriptomically intermediate between monocytes and mature lipid-associated macrophages (LAMs) and were consistent with a LAM precursor (pre-LAM). Pre-LAMs were spatially associated with early obesity crown-like structures (CLSs), which indicate adipose tissue dysfunction. Spatial data showed colocalization of ligand-receptor transcripts related to lipid signaling among monocytes, pre-LAMs, and LAMs, including *Apoe, Lrp1, Lpl*, and *App*. Pre-LAM expression of these ligands in early obesity suggested signaling to LAMs in the CLS microenvironment. Our results refine understanding of ATM diversity and provide insight into the dynamics of the LAM lineage during development of metabolic disease.

## Graphical abstract

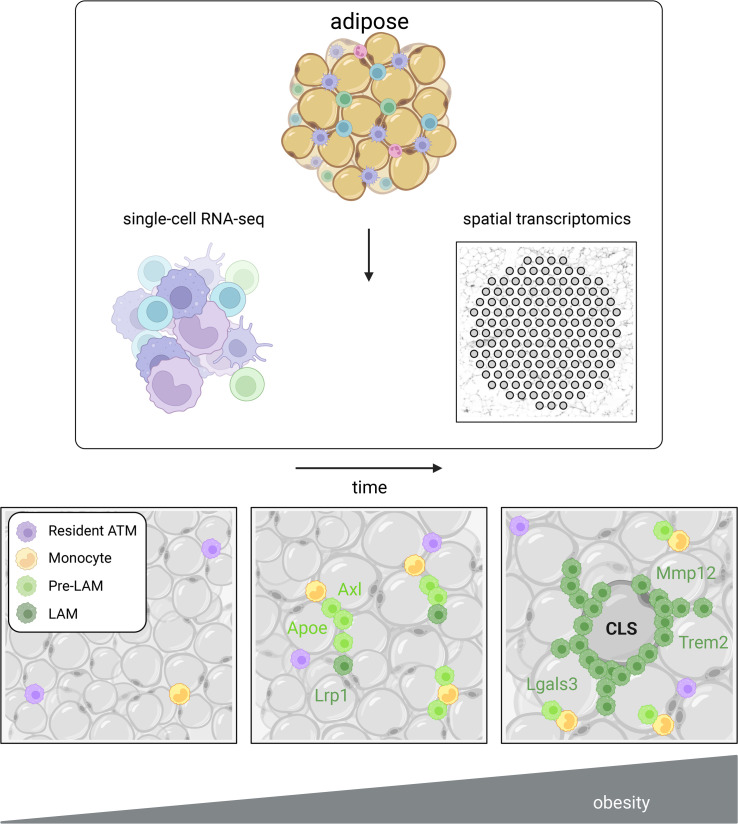

## Introduction

Obesity is associated with chronic inflammation and metabolic dysfunction in mice and humans (1–4). Increased metabolic demand requires remodeling of white adipose tissue (WAT) that results in changes to WAT structure and function (5, 6). Normal WAT function requires coordination between multiple cell types, including stromal vascular cells, immune cells, and adipocytes, which are the largest cellular constituent of WAT by volume (5, 7). In obesity, WAT composition is dramatically altered and cells undergo dynamic changes to their morphology and phenotype that culminate in adipocyte hypertrophy and cell death (5, 8). The dynamics of WAT immune cells during obesity are well documented, but the molecular mechanisms regulating immune and metabolic dysfunction and their spatial organization within WAT remain poorly understood.

Immune cells help maintain healthy WAT homeostatic function and participate in WAT remodeling in response to changes in metabolic demand. The hallmark of obesity-induced immune dysregulation is increased abundance and diversity of macrophages in WAT (9–11). Both tissue-resident macrophages and macrophages derived from recruited monocytes (MNs) acquire poorly understood activation states during obesity-induced WAT remodeling (9, 10, 12, 13). Changes in the macrophage transcriptional program are critical milestones in the development of insulin resistance, type 2 diabetes, and other metabolic disorders (9, 13) and persist after weight loss (11, 14, 15).

Previous single-cell studies have cataloged WAT cellular composition, thus refining our understanding of immune-cell phenotypes in obesity (7, 9, 10, 12). However, single-cell molecular profiling does not allow for analysis of the spatial patterning of tissue structure. Recent studies in humans have mapped single-cell genomic profiles onto spatial transcriptomics data to characterize spatial patterning of WAT cellular composition (5, 16). However, a spatial understanding of obesity-induced WAT-remodeling over the course of metabolic disruption is lacking.

Here, we sought to spatially contextualize immune-cell dynamics in early and chronic obesity. We sequenced single cells from murine WAT at different stages of diet-induced obesity and characterized transcriptional dynamics associated with the development of insulin resistance. To characterize the spatial context of obesity-driven immune-cell dysregulation, we mapped tissue-specific genomic signatures to the WAT landscape using spatial transcriptomics. We developed a network approach to analyze the spatial organization of immune dysregulation and used graph-theoretic measures to quantify changes to WAT structure.

We quantified the spatiotemporal dynamics of WAT macrophage infiltration and differentiation and identified cellular signaling mechanisms implicated in WAT remodeling. We describe diversity in the *Trem2*^+^ lipid-associated macrophage (LAM) phenotype, whose transcriptional profile, molecular signaling mechanisms, and spatial context suggest a critical role in the formation of crown-like structures (CLSs) in early obesity.

## Results

### Dynamic remodeling of adipose tissue is concurrent with glucose intolerance in early obesity.

**Figure 1:**
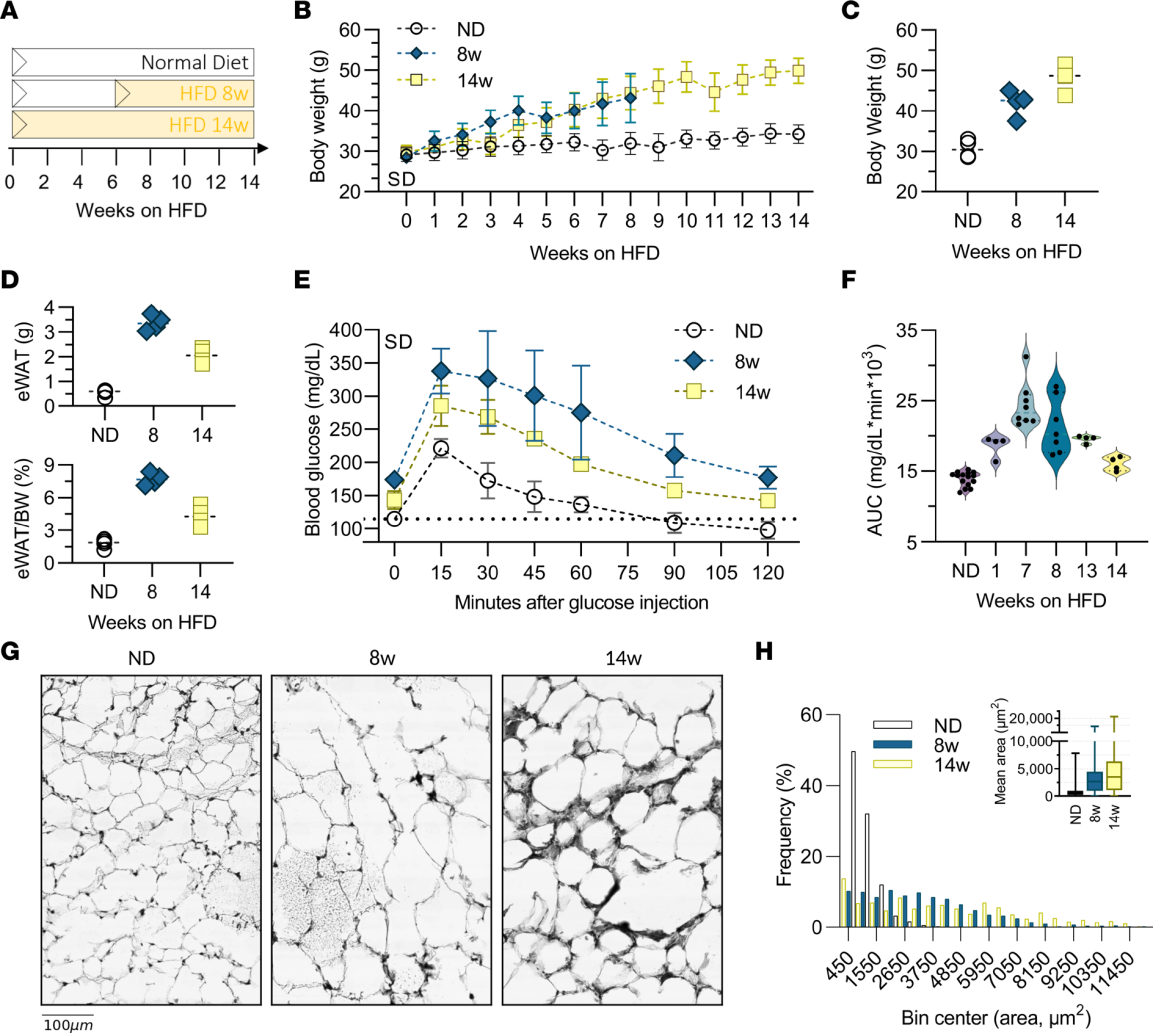
Diet-induced obesity and adipose tissue remodeling. (**A**) Time course for mice fed a 60% HFD for 8 weeks (8w) or 14 weeks (14w) versus ND controls. (**B**) Total BW by week on HFD. (**C**) Final BW at time of tissue collection. (**D**) eWAT weight (top) and eWAT as a percentage of BW (bottom). (**E**) Glucose measurements in cohorts 1 week prior to endpoint tissue collection. (**F**) GTT data showing AUC. (**G**) H&E-stained adipose tissue sections of eWAT from cohorts. (**H**) Frequency distribution and average adipocyte size in eWAT from cohorts. (**B**–**E**) For each group, *n* = 4. (**F**) For HFD-fed mice, *n* = 4–8 mice per group; for ND, n = 15 mice.

Our model of diet-induced obesity included mice fed a normal chow diet (ND) or a 60% high-fat diet (HFD) for 8 weeks as a model of early obesity during rapid adipose tissue growth (3, 17) or for 14 weeks as a model of chronic obesity (Figure 1A). HFD feeding increased BW and epididymal WAT (eWAT) mass as expected (Figure 1, B–D). Mean adipocyte area and frequency of large adipocytes increased at 8 and 14 weeks (Figure 1, G and H, and Supplemental Table 1; supplemental material available online with this article; https://doi.org/10.1172/jci.insight.171701DS1). Glucose tolerance tests (GTTs) showed increased AUC starting at week 1, with the largest AUC and variability at weeks 7 and 8 (Figure 1, E and F), suggesting development of insulin resistance.

### Single-cell profiling.

It is well established that obesity induces changes in adipose tissue immune cells (7, 9), including accumulation of adipose tissue macrophages (ATMs) that promote metabolic dysfunction (1, 2). However, the dynamics of these phenotypes remain incompletely understood. To examine immune-cell dynamics in early and chronic obesity, we performed single-cell RNA-Seq (scRNA-Seq) on CD45^+^ cells from eWAT of mice fed an ND or an HFD for 8 or 14 weeks (*n* = 4 per cohort).

**Figure 2:**
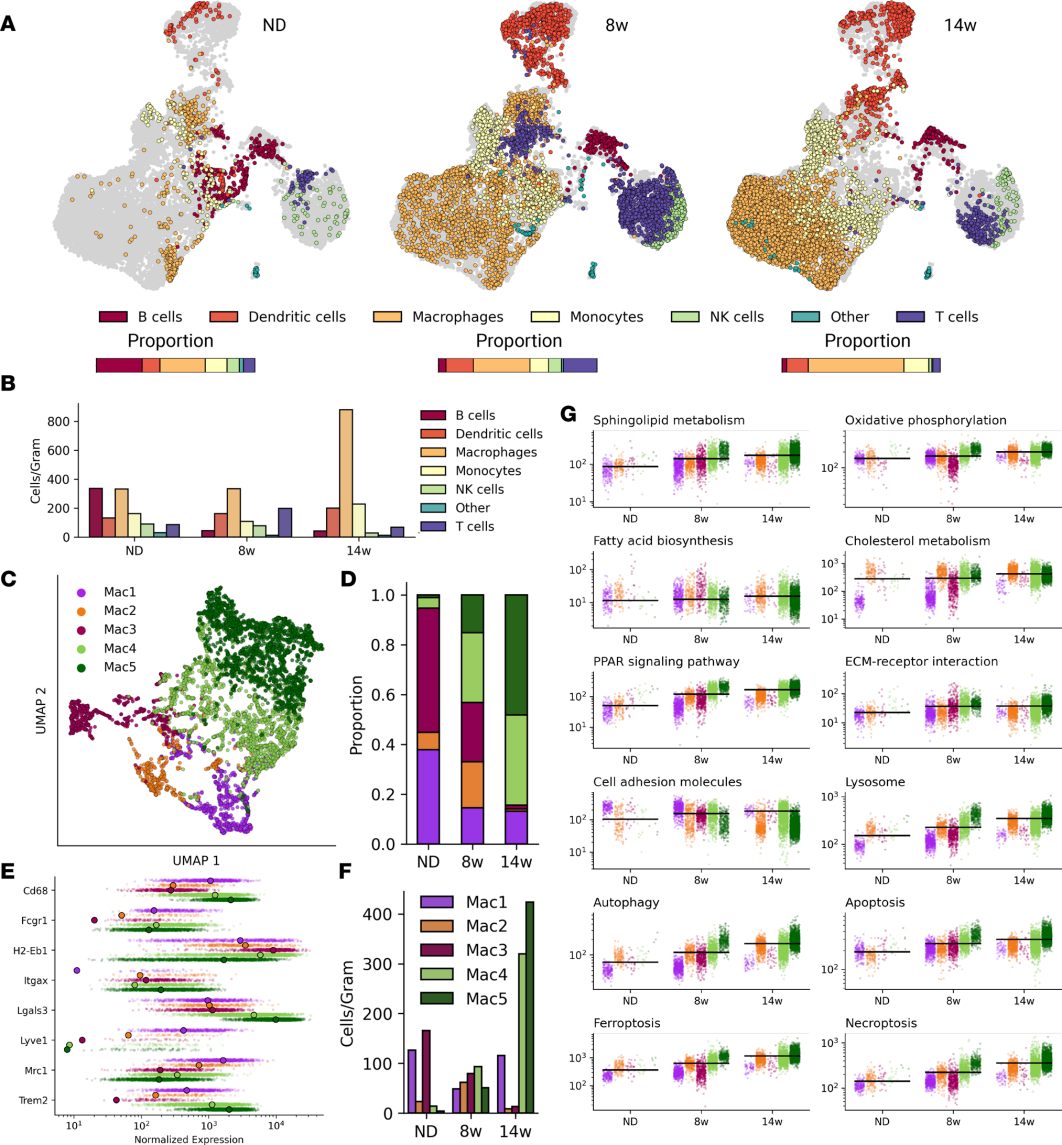
Single-cell data on macrophage phenotypes in obesity. (**A**) Immune-cell population changes over the course of diet-induced obesity. (**B**) The number of cells per gram of adipose tissue for each cell type in each diet condition. (**C**) UMAP visualization of ATM clusters from scRNA-Seq data. (**D**) Proportions of each ATM cluster at each time point. (**E**) Expression of key genes across ATM subtypes. Large points represent mean expression for the subtype. (**F**) ATM subtypes per gram of tissue sampled for each diet condition. (**G**) Changes in mean expression of genes in select Kyoto Encyclopedia of Genes and Genomes pathways in the macrophage subpopulations. Black lines represent mean macrophage expression of pathway genes in each diet condition. 8w, 8 weeks; 14w, 14 weeks; ECM, extracellular matrix.

Clustering and annotation of 13,820 single cells identified 6 broad immune-cell populations: MNs, T cells, B cells, DCs, ATMs, and NK cells (Figure 2A and Supplemental Table 2). Ab feature barcodes for select surface proteins that were used with scRNA-Seq confirmed immune-cell annotations (Supplemental Figure 1 and Supplemental Table 3). Annotations were additionally confirmed by comparison to cell type–specific gene expression profiles from public databases and published single-cell genomic data sets (Supplemental Figures 2–4).

Immune cells were then evaluated for changes across diet conditions. ATMs increased as expected with obesity, comprising 28%, 36%, and 60% of CD45^+^ cells in mice fed the ND, 8 weeks of the HFD, and 14 weeks of the HFD, respectively (Figure 2B and Supplemental Figure 5A). DC and MN populations also increased with HFD feeding, whereas the T cell population was highest at 8 weeks and decreased by 14 weeks of HFD feeding.

Altogether, our data capture expected eWAT immune-cell population dynamics in obesity progression and highlight myeloid cell accumulation in chronic obesity.

### ATM heterogeneity spans 5 subtypes across early obesity.

To define ATM heterogeneity, clustering was performed on ATMs from all diet conditions. Five ATM subclusters were identified corresponding to resident (Mac1), proinflammatory (Mac2, Mac3), and lipid-associated (Mac4, Mac5) macrophages (Figure 2C). Consistent with previous reports, Mac1 expressed *Lyve1*, *Timd4*, *Mrc1/Cd206*, and *Stab1* (Figure 2, C–E, and Supplemental Figures 6 and 7) (10, 18, 19).

Mac2 and Mac3 were identified on the basis of expression of genes encoding proinflammatory cytokines, including *Il1b*, *Tnf*, and *Il6*, and of low expression of efferocytosis markers (*Mertk*, *Axl*, *Cd163*, and *Trem2*) (Supplemental Figure 8). Among proinflammatory ATMs, Mac2s were enriched for the following additional proinflammatory genes: *Tnf, Il1b*, *Ccl2*, *Nlrp3*, and the M2 marker *Mrc1*. Mac3 had high expression of *Itgax/Cd11c* and antigen-presentation genes (*H2-Ab1*, *H2-Eb1*, *Cd74*) and was low in *Adgre1* (F4/80), suggesting an antigen-presenting ATM similar to that reported by Lantz et al. (20). Importantly, Mac3s were low in ATDC markers including *Zbtb46*, *Clec9a*, and *Cd24a* (Supplemental Figure 9) (10).

Finally, Mac4 and Mac5 ATMs emerged with HFD feeding and expressed genes consistent with LAMs, including *Trem2*, *Cd9*, and *Gpnmb* (Figure 2E) (9). Despite transcriptional similarities, Mac4 and Mac5 differed in magnitude of LAM marker expression (Figure 2G and Supplemental Figure 10).

Overall, these data highlight increased ATM diversity with HFD feeding.

### Lipid-associated ATMs overtake proinflammatory ATMs in chronic obesity.

Next, we examined ATM phenotype dynamics during HFD feeding. To assess broad changes in the ATM transcriptional program, we examined expression of gene sets associated with phenotypic shifts in macrophages. ATMs showed progressively increased gene expression related to lipid metabolism, migration, catabolism, and cell death (Figure 2G), supporting altered metabolism and survival processes in response to obesity.

We found that resident ATMs maintained a stable population over the course of HFD feeding (Figure 2, D and F). Proinflammatory macrophages were present in lean eWAT through 8 weeks of HFD feeding but decreased substantially after 14 weeks of HFD feeding (Figure 2, D and F, and Supplemental Figure 11, A and B). In contrast, LAMs emerged with HFD feeding and continued to accumulate in chronic obesity (Figure 2, D and F).

Given that other immune cells also have imbalanced subtypes in obesity and to provide additional context for ATM phenotypes during the time course, we further analyzed the single-cell data for subtypes of T cells, MNs, and DCs. Known subtypes that change in adipose tissue with obesity were identified, including decreased numbers of regulatory T cells, increased conventional T cells (T convs), and increased type 2 conventional DCs (Supplemental Figure 12) (9, 10). Extended analysis of T cells using classical marker gene sets also showed an increased proportion of T convs and increased *Mki67* expression among T cells, reflecting proliferation of an activated phenotype (Supplemental Figure 13)

Taken together, these data show that although the population of proinflammatory ATMs increases during adipose tissue hypertrophy, LAMs become the most prominent ATM subtype in chronic obesity.

### LAM subtypes form a monocytic lineage.

**Figure 3:**
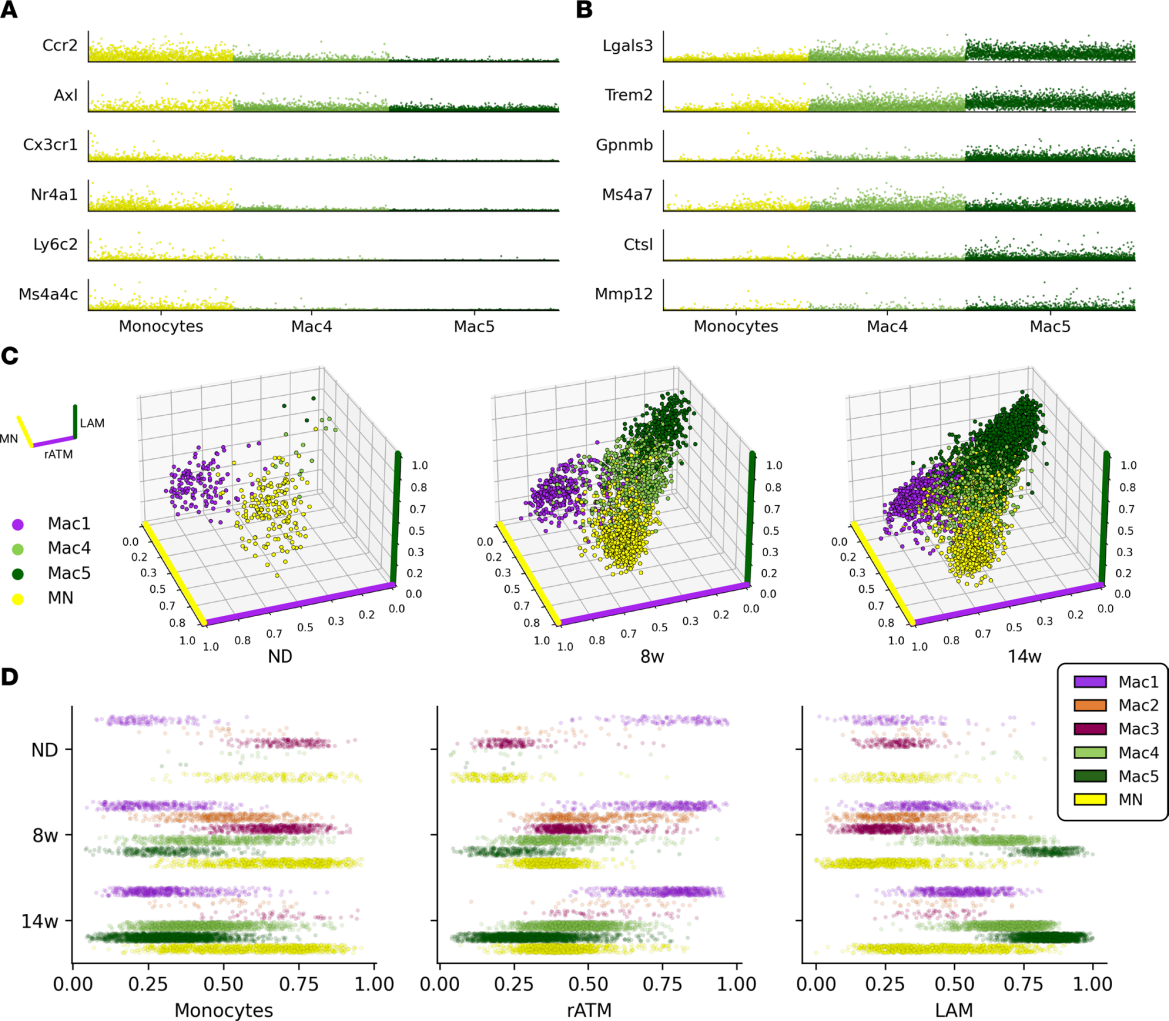
Emergence of the LAM phenotype. (**A**) Normalized expression of MN marker genes for key myeloid cell types. (**B**) Normalized expression of LAM marker genes for key myeloid cell types. (**C**) 3D profiling of MNs, resident ATMs (Mac1), and LAMs (Mac4, Mac5). Cell position represents simultaneous Pearson correlation with gene expression signatures derived from MNs (yellow axis), resident ATMs (rATM; purple axis), and LAMs (green axis). (**D**) Macrophage subtype correlations with MN, rATM, and LAM expression signatures for each diet condition. 8w, 8 weeks; 14w, 14 weeks.

We observed that between Trem2^+^ LAMs, Mac4 cells outnumbered Mac5 cells at 8 weeks (Figure 2, D and F), but Mac5 numbers were higher at 14 weeks of HFD feeding (Figure 2, D and F). Because LAMs are reported to be MN derived (9), we hypothesized that cells in the Mac4 cluster were in transition along a MN–LAM lineage. When we examined differentially expressed genes, 287 distinguished Mac4 cells and MNs, and 834 distinguished Mac5 cells and MNs (Supplemental Figure 6 and Supplemental Table 4), suggesting increasing divergence across MNs, Mac4 cells, and Mac5 cells. We then queried MNs, Mac4 cells, and Mac5 cells for expression of genes related to MN differentiation and macrophage maturity. The MN markers *Cx3cr1* and *Ly6c2* were decreased in the Mac4 cluster but were consistently higher in Mac4 compared with Mac5 (Figure 3A). Cells in the Mac4 cluster also showed intermediate expression of LAM marker genes *Lgals3*, *Trem2*, and *Ctsl* (Figure 3B). Mac4 cells also expressed *Ms4a7*, a marker of MN–macrophage differentiation, more highly than both MNs and Mac5 cells (21).

To further examine the hypothesis that Mac4 cells are pre-LAMs, we correlated them with resident ATMs (Mac1 in the ND-fed mice), early-onset MNs (in the ND and 8-week HFD groups), and chronic obesity LAMs (Mac5 in the 14-week HFD group) using the top 50 uniquely differentially expressed genes for each group. We found that Mac4 cells have intermediate correlation with the LAM and MN signatures. (Figure 3, C and D, and Supplemental Table 5).

Taken together, our data support that Mac4 cells are recently differentiated macrophages that are in process of acquiring the LAM phenotype.

### Spatial transcriptomics captures LAM dynamics in obesity.

The spatial context of ATM reprogramming within eWAT remains poorly understood. Thus, to establish the spatial dynamics of LAM emergence with obesity, we performed spatial transcriptomics on eWAT sampled from mice fed the ND or fed the HFD for 8 or 14 weeks (Supplemental Figure 14). We analyzed a total of 7424 tissue-capture spots across diet conditions.

**Figure 4:**
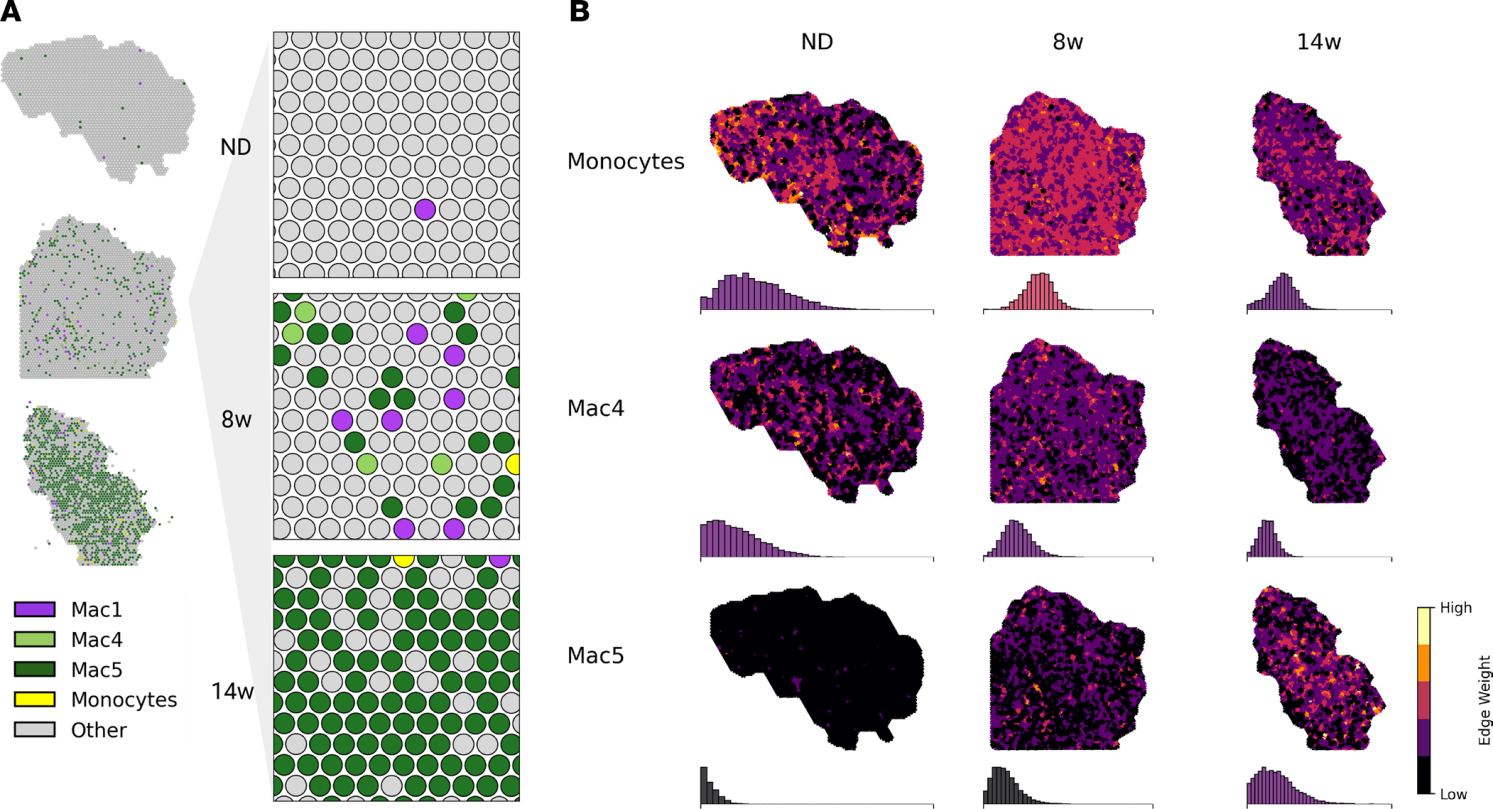
Spatial patterning of the MN-LAM lineage. (**A**) Overview of CARD-predicted cell-type proportions for myeloid cell types over the course of HFD feeding. (**B**) Spatial patterning of MNs, pre-LAMs (Mac4) and LAMs (Mac5) over the course of HFD feeding. Edge weights are the harmonic mean of CARD proportions for neighboring capture spots. Histograms show the distribution of edge weights for the whole tissue section and are colored according to the mean edge weight on the same color scale. 8w, 8 weeks; 14w, 14 weeks.

Immune-cell transcriptome profiles were mapped onto tissue-specific locations using conditional autoregressive-based deconvolution (CARD) (22, 23). We found strong emergence of the LAM phenotype across tissue spots in chronic obesity, consistent with our single-cell data (Figure 4, A and B, and Supplemental Figure 15, A and B). MNs also increased in spatial transcriptomics data in early obesity (Figure 4, A and B, and Supplemental Figure 15, A and B). Although pre-LAM spots were highest in early obesity, LAM spots were highest in chronic obesity (Supplemental Figure 15B). Furthermore, pre-LAMs and LAMs were highly spatially correlated at 8 weeks (*r* = 0.6) but not at 14 weeks (*r* = 0.2) (Supplemental Figure 16), suggesting that LAM dynamics are spatially coordinated. Taken together, these results support LAM accumulation in eWAT via differentiation from circulating MNs.

### LAM networks are hubs of cell death.

**Figure 5:**
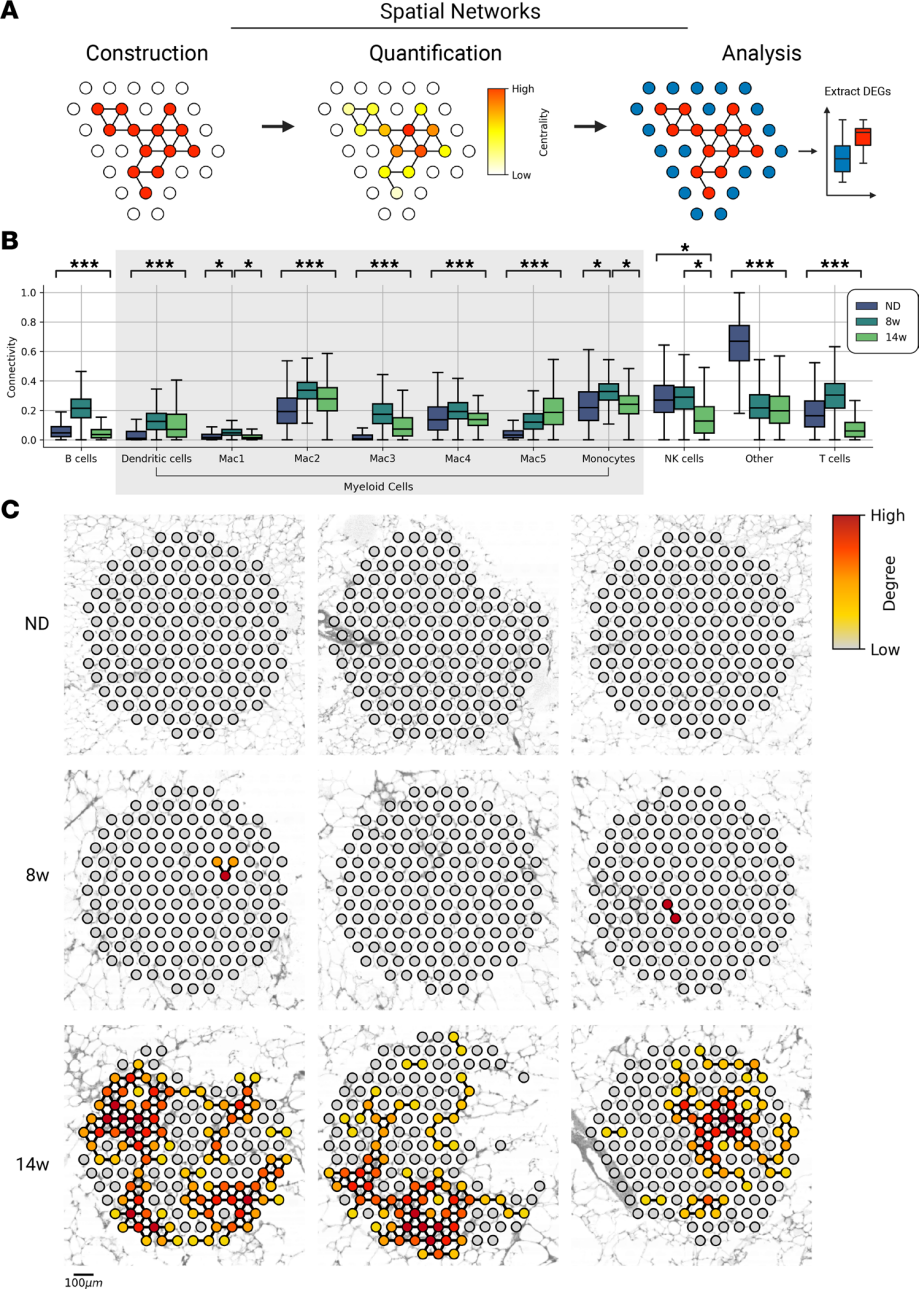
LAM networks are hubs of cell death. (**A**) Workflow schematic. Network models are defined on the basis of properties of neighboring tissue spots. Analysis of network structure reveals principals of tissue organization. Differential expression analysis may be used to characterize the transcriptional signature of niches. (**B**) Connectivity of tissue-wide networks for all immune-cell types over time. Connectivity is the distribution of network edge weights, defined as the harmonic mean of CARD-predicted proportions between neighboring spots. ***P = 0.01 by Student’s *t* test for comparison between each time point (i.e., ND vs. 8 weeks [8w], 8w vs. 14 weeks [14w], and ND vs. 14w); *P ≤ 0.01 for specific comparison. (**C**) Nine randomly sampled 150-node networks based on LAM signature (Mac5) over time. DEG, differentially expressed gene.

LAMs are associated with development of CLSs, which, in turn, are correlated with development of insulin resistance (13, 24, 25). CLS are well studied (8, 26), though a spatiotemporal understanding of the drivers of CLS formation is lacking. We observed CLSs as early as 8 weeks, which prompted us to characterize the transcript patterns associated with early CLS formation. We developed cell type–specific network models based on spatial gene expression patterns and used the models to understand the dynamics of adipose tissue organization in obesity (Figure 5A).

Network models represent local tissue regions where a given cell type is highly localized. In the networks, nodes represent tissue-capture spots and edges represent interactions between adjacent nodes. Edges were defined by the harmonic mean of CARD-predicted proportions between all adjacent pairs of nodes for a given cell type. The structural properties of the cell-type networks were quantified using graph-theoretic measures, which, in turn, revealed properties of tissue organization (Figure 5A) (27).

Network models showed higher local concentrations of adaptive immune cells (i.e., B cells, T cells) in the week 8 group than in lean tissue or the week 14 group, which coincided with the emergence of proinflammatory ATMs (Figure 5B and Supplemental Table 6). In addition, proinflammatory Mac3 and T cells were spatially correlated at 8 weeks (*r* = 0.6), in contrast to low Mac1 (*r* = –0.1) and Mac2 (*r* = 0.2) spatial correlation with T cells (Supplemental Figure 16). These results suggest T cell activation, which is consistent with the emergence of T conv at 8 weeks (Supplemental Figure 12).

In contrast, local LAM concentrations increased monotonically over the course of HFD feeding, further supporting that ATM reprogramming toward the LAM phenotype is spatially coordinated. To further investigate LAM spatial patterning, we randomly sampled tissue spots from all 3 diet conditions and constructed 150-node networks around the sampled spot (Figure 5C). As expected, high local LAM concentrations were absent in lean tissue (Figure 5, B and C). With HFD feeding, LAM concentration increased (Figure 5C and Supplemental Figure 17). We then performed differential expression analysis between regions of high and low LAM concentrations and found that regions of high LAM concentrations were enriched in genes related to phagocytosis, autophagy, and cell death, including *Ctsl*, *Ctss*, *Lamp1*, *Ctsd*, and *Ctsb* (Supplemental Figure 18). Altogether, these results identify spatially coordinated accumulation of LAMs that are engaged in clearance of excess lipids and dead adipocytes.

### LAM networks map onto histologically identified CLSs.

**Figure 6:**
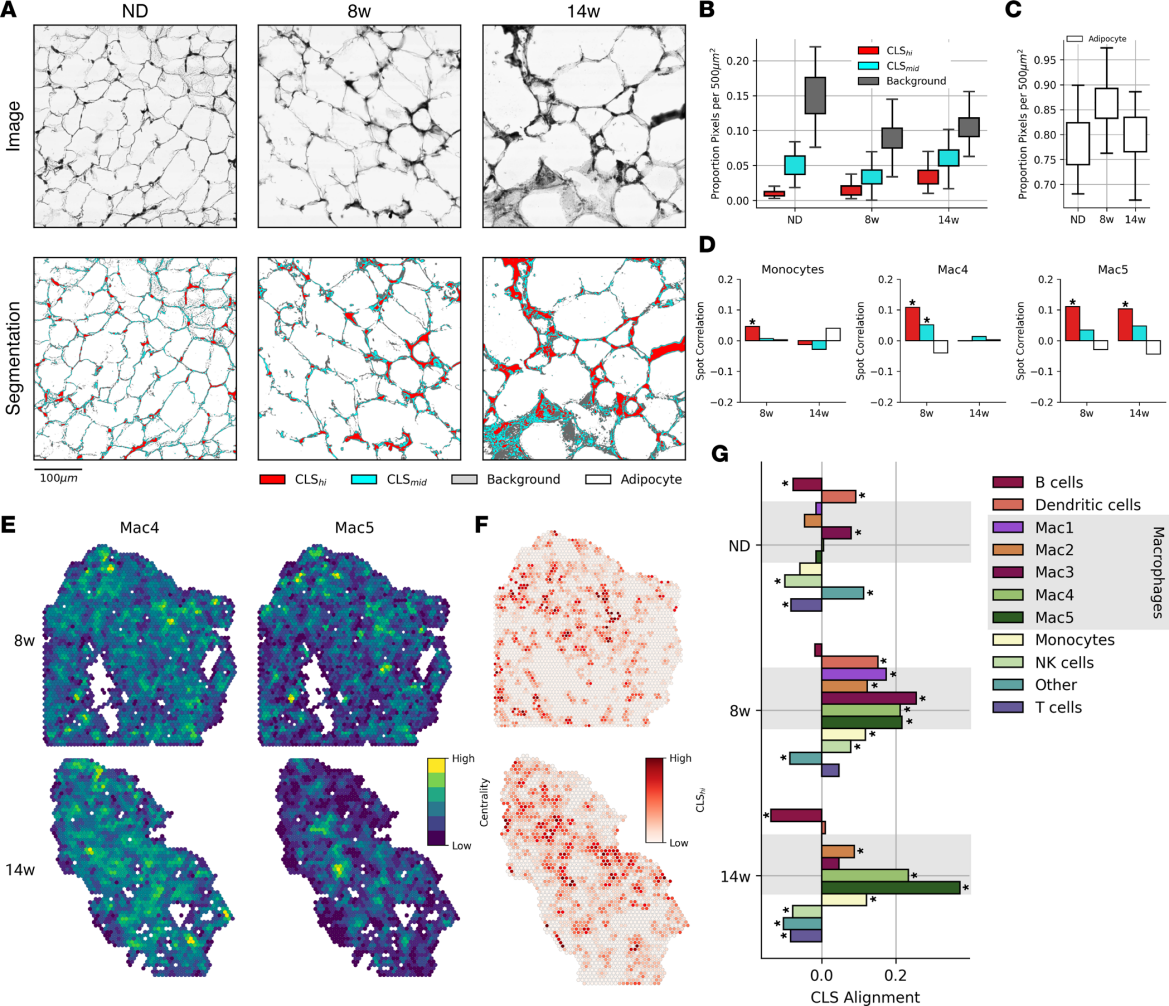
Histological quantification of CLSs. (**A**) H&E-stained images captured during spatial transcriptomics library preparation (top) and segmentation results quantifying CLSs (bottom). (**B**) Segmentation class label proportions of 100 randomly sampled 500 μm regions from each diet condition. (**C**) Adipocyte area from images regions in **B**. (**D**) Spot correlation between myeloid cell-type proportions and segmentation results from a 150 μm region around each capture spot. Spots with read counts below the 0.05 quantile were removed. *P ≤ 0.01 by Pearson correlation. (**E**) Spot importance in global cell-type networks (eigenvector centrality) in HFD feeding conditions. Eigenvector centrality highlights regions of densely localized cells in the tissue. (**F**) CLS_hi_ segmentation results in 150 μm regions around each capture spot at 8 weeks (8w) and 14 weeks (14w). (**G**) CLS alignment represents the Pearson correlation between CLS_hi_ segmentation results and cell type–specific eigenvector centrality for each diet condition. *P ≤ 0.01 by Pearson correlation. Spots with read counts below the 0.05 quantile were removed.

CLSs are defined by an accumulation of fibrotic and necrotic material from dead or dying adipocytes and ATMs (8, 26). To determine the degree to which the LAM network was spatially aligned with CLSs, we first developed an image segmentation algorithm to classify CLS regions from H&E-stained images captured in parallel with spatial transcriptomics data (Figure 6A). The algorithm identified CLS_hi_ and CLS_mid_ regions of fibrotic and necrotic material that increased with obesity (Figure 6B). Adipocyte area increased with HFD feeding but decreased between 8 and 14 weeks as a higher proportion of the area was represented by CLSs and regions of immune infiltration (Figure 6C). We then aligned CLS regions with spatial transcriptomics data and found significant colocalization of LAMs with CLSs in both early and chronic obesity (Figure 6D). In contrast, MNs and pre-LAMs colocalized with CLS regions only in early obesity (Figure 6D).

Beyond correlation, we sought to characterize the physical organization of immune-cell types within eWAT and their relationship to CLSs. We used eigenvector centrality, a global measure of nodal importance in a network, to quantify cell type–specific structure within the tissue (27). We then correlated per-spot centrality for each immune cell–type network with per-spot CLS prevalence (Figure 6G). We found that critical hubs of innate immune cells aligned with early CLSs in week 8 (Figure 6G). Central nodes in pre-LAM and LAM networks aligned with CLSs both in early and chronic obesity (Figure 6, E–G). In contrast, adaptive immune-cell types (i.e., B cells, T cells) exhibited negative correlation with CLSs in all diet conditions.

Taken together, these results capture the dynamic, large-scale reorganization of immune cells in early obesity and the spatial concentration of LAMs in CLS regions in chronic obesity.

### Myeloid signaling shapes nascent CLSs.

Given the early presence of CLSs and reorganization of myeloid cell types in week 8, we sought to characterize intracellular signaling during formation of CLSs. Therefore, we quantified spatially colocalized expression of ligand-receptor (LR) pairs throughout eWAT and within the MN–LAM lineage.

**Figure 7:**
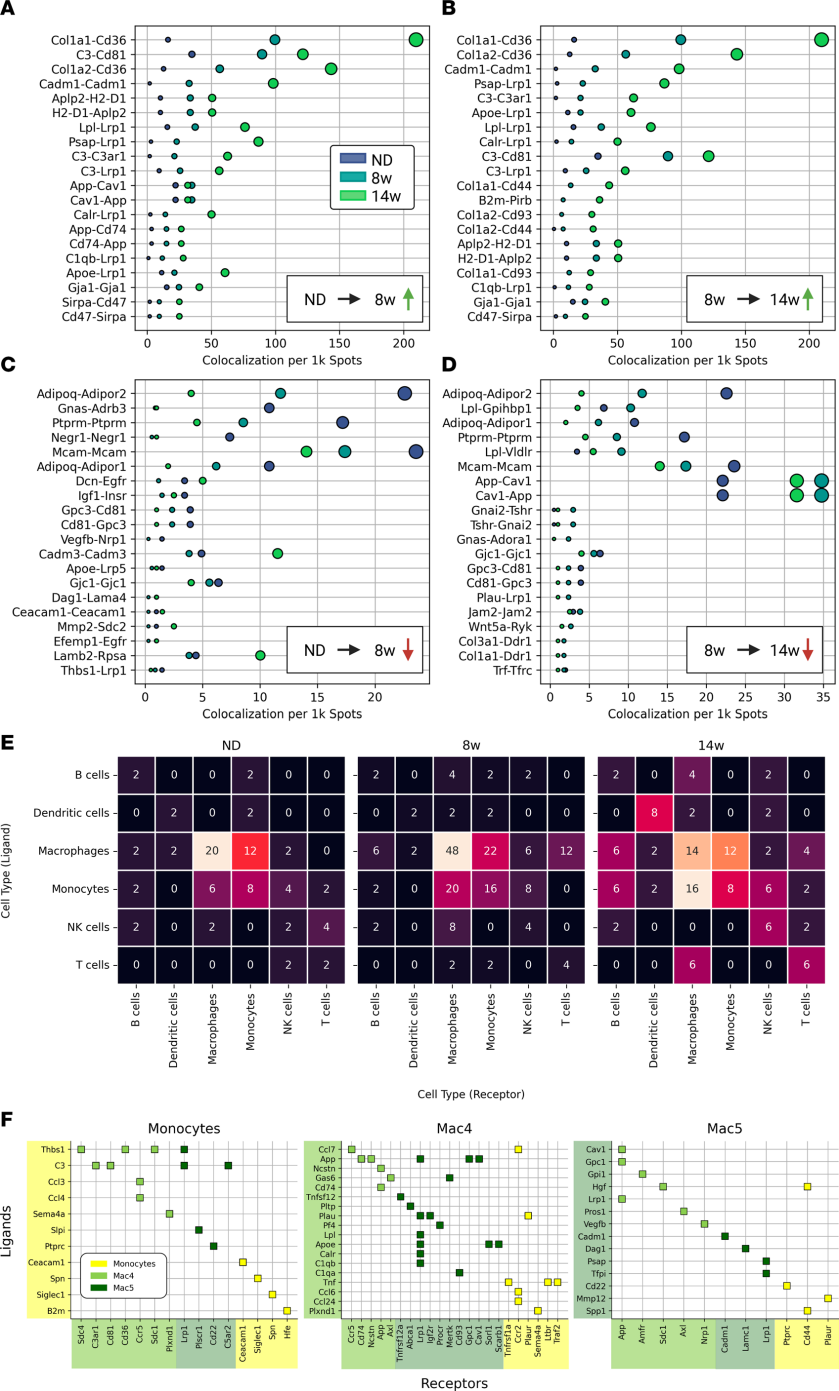
eWAT LR signaling dynamics. (**A**) LR pairs with most increased colocalization during the first 8 weeks (8w) of HFD feeding. Dot sizes are LR colocalization per 1000 (1k) capture spots (same as *x* axis) and dot colors indicate diet condition. (**B**) LR pairs with most increased colocalization during the last 6 weeks of HFD feeding. (**C**) LR pairs with most decreased colocalization during the first 8 weeks of HFD feeding. (**D**) LR pairs with most decreased colocalization during last 6 weeks of HFD feeding. (**E**) Counts of colocalized cell type–specific LR pairs with log fold change > 1 in each diet condition. (**F**) Differentially expressed myeloid LR pairs using Wilcoxon rank-sum test (α = 0.05, Bonferroni corrected) with nonzero colocalization in spatial data. 14w, 14 weeks.

We first cataloged tissue-wide changes in LR expression. We identified the LR pairs that increased in early obesity and chronic obesity (Figure 7, A and B) and the LR pairs that decreased in early and chronic obesity (Figure 7, C and D). As expected, global LR analysis revealed increased metabolic activation (*Lrp1*, *Lpl*, *App*, *Apoe*), regulation of cellular migration (*Adipoq*, *Igf1*, *Thbs1*, *Apoe*), regulation of tissue remodeling (*Cola1*, *Cola2*), and regulation of immune response (*Cd36*, *Cd81*, *C3*) (Figure 7, A–D, and Supplemental Figure 19) as predominant biological processes associated with obesity-induced eWAT remodeling.

Next, we identified colocalized LR pairs with cell type–specific expression (log2 fold change > 1; Supplemental Figure 20). Detection of LR pairs between myeloid cell types increased with obesity (Figure 7E). To identify the myeloid-specific signaling that may contribute to the emergence of CLSs, we investigated LR pairs that were both differentially expressed in a myeloid cell subtype and colocalized with one another in the spatial transcriptomics data (Figure 7F). Pre-LAMs expressed multiple ligands for LAM receptor *Lrp1*, including *App*, *Plau*, *Lpl*, *Apoe*, *Calr*, and *C1qb*. Additionally, pre-LAMs expressed ligands *App*, *Plau*, and *Apoe* that had multiple receptors throughout the MN–LAM lineage.

Thus, we identify a set of signaling molecules expressed in early obesity along the MN–LAM lineage that influence the nascent CLS microenvironment.

## Discussion

Changes in adipose tissue myeloid cells persist with weight loss and affect future response to overnutrition (11, 14), highlighting the need to better understand the mechanisms that promote adipose tissue dysfunction. Our study elucidates ATM phenotype dynamics in their spatial context in early and chronic obesity by combining single-cell RNA-Seq, spatial transcriptomics, and imaging over time.

Our work supports increased phenotypic diversity in ATMs with obesity that is consistent with other single-cell work (9, 10, 26, 28). Our data captured a dramatic increase in ATMs that were phenotypically distinct from resident ATMs in lean tissue (Figure 3), and ATMs overall showed metabolic and catabolic activation in obesity (Figure 3A). Increases in numbers of proinflammatory macrophages were evident in early obesity, consistent with previous reports (29). However, we show that the LAM phenotype became dominant among ATMs in chronic obesity (Figure 2, D and F) (7, 9, 13). These data are consistent with other work demonstrating that ATMs acquire nonclassical activation states in obesity and accumulate internal lipids (3, 13, 28, 30, 31). We note that increased lipid-uptake and proinflammatory phenotypes are not necessarily mutually exclusive.

LAMs are reported to be antiinflammatory, tissue-remodeling macrophages that are highly metabolically active; their transcriptional signature is associated with phagocytosis and endocytosis (12), and they have elevated expression of markers such as *Trem2*, *Lgals3*, and *Ctsl* (9). Although there is no consensus, a growing body of research supports the hypothesis that LAM function is predominantly protective (9, 12, 32–35). LAMs are thought to mitigate the adverse effects of obesity-induced adipose tissue remodeling through clearance of dead adipocytes (9, 12, 32–35). Trem2 KO studies demonstrated that Trem2 was required for the emergence of the LAM phenotype and the formation of CLSs, and that ablation of Trem2 leads to increased levels of adipocyte hypertrophy and death (9, 32).

Our data agree with these findings and additionally identify a population of MN-derived pre-LAMs as a closely related precursor to LAMs, which is supported by both previous lineage-tracing experiments (9) and by RNA velocity analysis of murine scRNA-Seq data (10) (Figure 3). Significant appearance of pre-LAMs precedes accumulation of LAMs and coincides with initial formation of CLSs. Spatial analyses further support pre-LAM localization to CLSs in early obesity and suggest pre-LAM signaling through App, Apoe, Lpl, and Lrp1 as drivers of CLS formation.

These molecules implicate disruption of lipid-processing pathways in development of tissue dysfunction. Dysregulated lipid processing is associated with oxidative and ER stress that alters cell survival and macrophage phenotype (13, 36–38), which are, in turn, hallmarks of disease progression in type 2 diabetes and Alzheimer’s disease (39, 40). For example, disease-associated microglia in Alzheimer’s disease bear similarity to LAMs and show increased expression of Trem2 ligands Lpl and Apoe (39–42).

We note that the spatial transcriptomics data included 1 tissue section per diet condition with the exception of lean adipose tissue, which had an additional replicate. All measures examined were consistent between lean replicates. Additionally, we used thresholds for the number of spots required for a given observation, such as for colocalized ligand–receptor pairs. The spatial transcriptomics data also presented a bioinformatic challenge related to low read depth (Supplemental Figure 21). Matched single-cell data aided in this analysis, and we used an inference approach to improve identification of cell types at each spot (22). Improvements in unbiased spatial technology, as well as more targeted transcript and protein-level studies (18), will provide additional resolution on the cell types and signaling identified here. Data were only collected from male mice and thus provide no comparisons based on sex, which may be a factor in adipose tissue inflammation and development of insulin resistance in obesity (43, 44).

### Conclusions.

Our data revise current understanding of ATM phenotypic shifts in obesity. We identify important milestones in MN–LAM development and provide spatial context for myeloid signaling that is implicated in metabolic dysfunction. Our study provides clarity on the cell types and signaling involved in CLS formation and accumulation, including the spatial dynamics of LAM development in obesity.

## Methods

### Animals.

C57BL/6J mice were used for all experiments (Jackson Laboratories; catalog 000664). Male mice were fed ad libitum a control ND (13.4% fat; LabDiet, catalog 5L0D) or an HFD (60% calories from fat; Research Diets, catalog D12492) for the indicated amount of time starting at age 9 weeks. Animals were housed in a specific pathogen-free facility with a 12-hour light/12-hour dark cycle and given free access to food and water except for withdrawal of food for temporary fasting associated with GTTs.

### GTTs.

For GTTs, starting 4 hours into the light cycle, mice were fasted with ad libitum access to water for 6 hours in clean cages. A 100 mg/mL d-glucose (Sigma; catalog G7021) solution was prepared in sterile –/– Dulbecco’s PBS (DPBS) and injected at 0.7 g/kg BW. AUC calculations were performed using the log trapezoidal method.

### Cell isolation and enrichment.

Stromal vascular cells (SVCs) were collected from adipose tissues as indicated by Muir et al. (3). After cardiac perfusion, adipose tissues were collected, minced finely to 3–5 mm pieces, and added to ice-cold HBSS plus Ca/Mg. Up to 1.5 g of tissue per sample was digested in 10 mL of 1 mg/mL collagenase II (Sigma; catalog C68850) in HBSS plus Ca/Mg at 37°C for 45 minutes with vigorous shaking. Digests were filtered through buffer-soaked 100 micron cell strainers and centrifuged at 300*g* at 4°C to pellet SVCs. SVCs were enriched for CD45^+^ immune cells using MojoSort Mouse CD45 Nanobeads (Biolegend; catalog 480027) following the manufacturer’s protocol. Briefly, SVC pellets were resuspended in 1 mL of MojoSort Buffer, pooling the 4 samples from each cohort (ND group, and 8-week and 14-week HFD groups) into a single, respective cohort tube, then filtered through a 70 μm cell strainer and placed in 5 mL polypropylene tubes. After addition of nanobeads, samples were sequentially processed for magnetic separation. Three magnetic separations in total were performed on the labeled fractions for increased purity. Final cell suspensions were filtered through 40 μm pipette tip filters. Cell viability was >80% with <15% aggregation.

### Library preparation.

CD45^+^ SVCs were feature barcoded using TotalSeqB (Biolegend) Abs. The following Abs were used: CD4 (catalog 100573), CD11b/Itgam (101273), CD19 (115563), F4/80/Adgre1 (123155), CD3 (100257), and Mac-2 (125425). Library preparation was performed by the University of Michigan Single-Cell Sequencing Core using the 10x Genomics Chromium Single Cell Kit (3′ v3; catalog 220103/PN120262). For single-cell transcript data, 100 million reads from up to 5000 cells were collected, and 25 million reads were collected from up to 5000 cells were collected for feature barcoding data.

For spatial transcriptomics, within 30 minutes of cardiac perfusion, eWAT samples that were contralateral to those used for scRNA-Seq were carefully dissected for placement in cryomolds. For consistent sampling, fat pads were aligned anterior (tip) to posterior (proximal to testis), and a razor blade was used to cut a strip from just anterior to midline to just posterior to the tip. Samples were presoaked in ice-cold OCT compound (VWR; catalog 25608-930) and placed in biopsy cryomolds (VWR; catalog 25608-922) with fresh OCT compound, rapidly frozen by immersion in isopentane cooled using liquid nitrogen, and kept on dry ice or at –80°C until sectioning. Fresh tissue sections were cut at 10 μm after 20 minutes of equilibration in a cryochamber set to –26°C or below with specimen arm at –40°C. Sections were placed onto the Visium Spatial Gene Expression slide and subsequent processing and library preparation were performed by the University of Michigan In Vivo Animal Core pathology laboratory and the Advanced Genomics Core according to the manufacturer’s protocol (10x Genomics; catalog PN-1000184).

### Tissue histology and immunostaining.

H&E and immunostaining were performed in the Unit for Laboratory Animal Medicine In Vivo Animal Core pathology laboratory at the University of Michigan. After fixation for 48 hours in 10% neutral buffered formalin, tissues were trimmed, cassetted, and processed to paraffin in an automated tissue processor (Tissue-Tek; Sakura). Processed tissues were embedded in paraffin and sectioned at 4 μm on a rotary microtome (Leica Biosystems). Tissues were mounted on glass slides and stained with H&E using routine protocols on an automated histostainer (Leica ST5010 Autostainer; Leica Biosystems), followed by coverslipping.

### Data processing.

scRNA-Seq files were processed using the 10x Genomics CellRanger (version 4.0.0) pipeline. The resulting filtered matrices were analyzed using the Python library SCANPY (45). Briefly, we filtered out cells that did not express at least 500 genes and genes that were not expressed in at least 10 cells, resulting in 13,820 cells and 31,053 genes across all diet conditions (*n* = 1,261 ND cells; *n* = 6,123 8-week HFD cells; and *n* = 6,436 14-week HFD cells). We normalized read counts per cell after filtering. Spatial sequencing data were processed using the 10x Genomics SpaceRanger (version 1.0.0) pipeline with mouse reference GRCm38, and resulting feature-barcode matrices were loaded into SCANPY (45) for further analysis. We filtered out capture spots that expressed fewer than 5 genes from all subsequent analysis. We normalized read counts per capture spot after filtering.

### scRNA-Seq clustering and visualization.

Clustering was performed on cells from each time point independently using Algorithm 1 (Supplemental Algorithm 1). Preprocessing and clustering were performed using Python and the single-cell gene expression package SCANPY (45). scRNA-Seq data were normalized and log-transformed before dimension reduction using principal component analysis with *r* = 50. We constructed the similarity matrix A using *k* = 9 neighbors and Euclidean distance prior to clustering with the Leiden clustering method (46) with resolution parameter = 0.95. This analysis resulted in 18 clusters in the ND group, 25 in the 8-week HFD-fed mice, and 20 in the 14-week HFD-fed mice. Visualization of data was performed using uniform manifold approximation and projection (UMAP) (47). Dimensionality was reduced using principal component analysis (*r* = 10) on the combined set of genes with nonzero expression at all 3 time points. Cells were passed to UMAP with the following parameters: *n* neighbors = 50, minimum distance = 0.25, and metric = ‘euclidean’.

### scRNA-Seq cell-type annotation.

Annotation of cell types after clustering was performed using ranked expression of cell type–specific mouse markers genes from PanglaoDB (48). The top 50 most unique marker genes were used for each cell type, sorted by their ubiquitousness index. Each cluster was assigned to a cell type on the basis of the maximum mean rank of marker genes among the differentially expressed genes for that cluster. A small set of 165 CD45^+^ cells was also identified that did not align with major immune-cell populations but partially aligned with stromal cells and preadipocytes (Supplemental Figure 3). This population was excluded from subsequent analyses. We performed differential expression analysis on clusters and sorted genes by their Student’s *t* test statistic computed using the scanpy.tl.rank genes groups() function with method = ‘t-test’.

### Deconvoluting spatial data.

We used a CARD model (https://github.com/YMa-lab/CARD/commit/469704f6031d3286bfedccb037c9b041c72b7892) to spatially deconvolute cell-type signatures of our data and estimate the strength of cell-type proportions across tissue-capture spots (22). CARD was chosen over other deconvolution methods for its ability to leverage nearby spatial information during cell-type proportion estimation using a conditional autoregressive modeling assumption, which imposes spatial correlation structure on the outputs. Briefly, each single cell was annotated for cell type and scRNA-Seq count matrices, and spatial transcriptomics count matrices were structured according to CARD documentation. Deconvolution was performed using createCARDObject() with parameters minCountGene = 10, and minCountSpot = 20. Outputs were stored as tabular files for downstream analysis. CARD estimates the cell-type proportions for *k* cell types defined given *g* genes at *n* tissue spots using the following nonnegative matrix factorization model:

X = BV^T^ + *E* (Equation 1)

where *X* ∈ *R*^g×n^ represents the spatial transcriptomics data matrix, *B* ∈ *R*^g×k^ is a matrix of aggregate cell-type signatures derived from the scRNA-Seq data, *V* ∈ *R*^n×k^ is a matrix of cell-type proportions at each tissue spot, *E* ∈ R^g×n^ is a normally distributed error matrix, and ^T^ denotes the matrix transpose. For further details, see Ma and Zhou (22).

### Macrophage continuum analysis.

A linear model was used to quantify cells along a user-defined continuum as in Li et al. (28, 49). The procedure from Li et al. (28) is generalized in Algorithm 2 (Supplemental Algorithm 2). Briefly, we used ordinary least squares (OLS) to linearize the correlation between 2 states of interest in a given cell population (e.g., ATM–LAM or MN–LAM). We quantified each cell’s position relative to the states of interest by computing the distance between the cell and each state along the OLS solution. We defined a gene set using differential expression between the 2 states with a Bonferroni correction for multiple tests to α = 0.05 ( 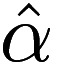
= 1.65 × 10^6^) and chose top genes for each pole, ranked by their fold change.

### LR colocalization.

Our list of mouse LR pairs was based on Baccin et al. (50). We defined colocalization as the simultaneous expression of ligand *l* and receptor *r* at a given tissue-capture spot *t*. The colocalization strength, or *l* and *r* at *t*, was quantified using the geometric mean of normalized expression, as follows:


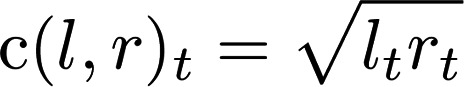
  (Equation 2) 

where *l_t_* and *r_t_* are the expression of *l* at *t* and *r* at *t*, respectively. By using the geometric mean, we ensure that c(*l,r*) = 0, where either *l_t_* = 0 or *r_t_* = 0. LR pairs are said to be colocalized wherever *c*(*l,r*)*_t_* > 0. Time-dependent colocalization between LR pairs was taken as a necessary, but not sufficient, condition in determining possible signaling pathways. We computed the proportion of spots where *l* and *r* were localized and normalized the proportion to 1000 spots to account for differences in tissue-section sizes.

### Network models.

We aim to construct a network model that preserves spatial relationships in tissue structure. Let G be a finite, simple, and undirected graph with node set *V* (G) = {1,2*...,n*} and edge set *E*(G) C *V* (G) × *V* (G). Let *e_ij_* be an edge between node *i* and node *j*. The *n* nodes of G are chosen from the set of tissue-capture spots from the spatial transcriptomics data matrix. Thus, each node *i* has a specified spatial position in a 2-dimensional Euclidean plane, *p_i_* 2R^2^. Edges are defined between nodes as a function of (a) their Euclidean distance and (b) their nodal properties determined by the biological question of interest. In the simplest case, we may define a radius *r*, which is the maximum physical interaction distance between 2 nodes. The strength of the relationship between node *i* and node *j* is encoded in the edge weight *w_ij_*. Edge weights are defined by a function, *f:* *V* (*G*) × *V* (*G*) → *R*.


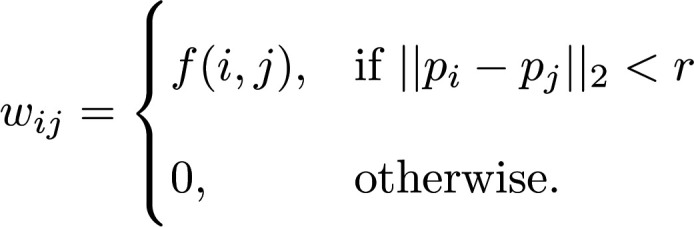
  (Equation 3)

A network defined this way captures the spatial patterning of *f* in the local neighborhood constrained by *r*. It is also useful to define the weighted adjacency matrix of G to be the n × n matrix A(G) with rows and columns indexed by V (G). We will denote A(G) as A and the entry (i, j) of A as A(*i,j*) = *a_ij_*. The weighted adjacency matrix otherwise may be defined as follows:


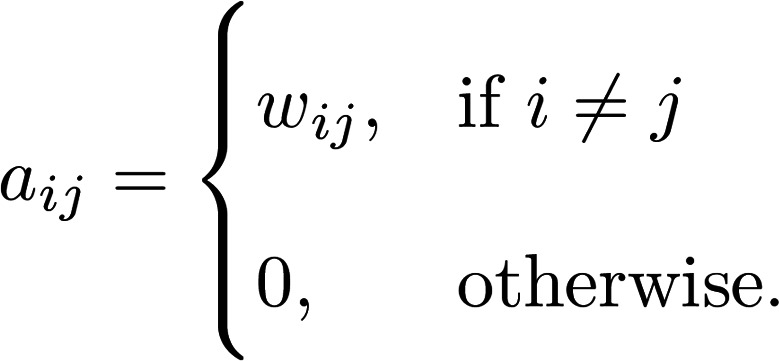
  (Equation 4)

For example, we define LAM networks on the basis of the harmonic mean of Mac5 CARD estimated proportions over neighboring tissue spots (22). In this case, the choice of the harmonic mean is based on the interpretation of CARD outputs as proportions of the tissue spot explained by a given cell-type signature (22). Let *m_i_* be the proportion of Mac5 cell type at tissue spot *i*:


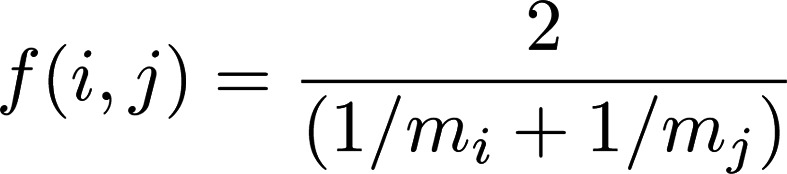
  (Equation 5)

The concept of network centrality is motivated by identification of “important” nodes of a network (27). We focus on 2 measures of network centrality: degree centrality (Equation 6) and eigenvector centrality (Equation 7). Degree centrality is a “local” measure of connectivity, whereas eigenvector centrality is a “global” measure of centrality. Let c*^d^_i_* denote the degree centrality of node *i*. Degree centrality is the sum of all the edge weights of node *i*, as follows:


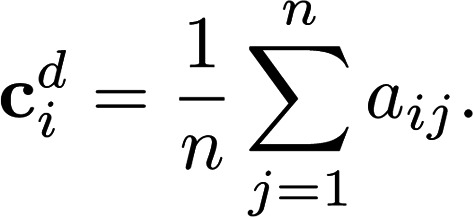
  (Equation 6)

The eigenvector centrality of each node, defined here up to a scale factor, is proportional to the sum of the eigenvector centralities of its neighbors, that is:


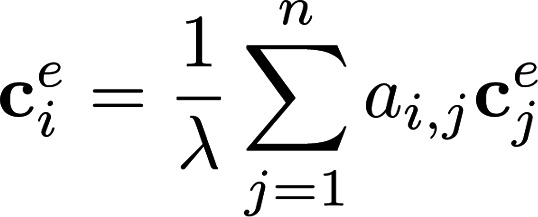
  (Equation 7)

where c^e^ is an eigenvector of A and is the corresponding eigenvalue. The centrality is taken to be an eigenvector that corresponds to the largest eigenvalue of A.

### Adipocyte sizing.

Images of H&E-stained adipose tissue were analyzed for adipocyte size using the Python package *skimage* (51). Briefly, images were converted to grayscale and subjected to an unsharp masking filter with the following parameters: amount = 75 and amount = 100. Filtered images were filtered again using a median filter with default parameterization followed by morphological reconstruction using method = ‘erosion’ to enhance contrast between neighboring cells. Finally, images were filtered using a Gaussian kernel with sigma = 3. Processed images were thresholded at the 25th percentile before segmentation using the watershed method. Properties of each segmented cell were obtained using measure.regionprops(). We computed the circularity *C* of all segmentation using Equation 8:


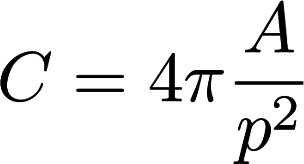
  (Equation 8)

where *A* is the estimated area and *p* is the estimated perimeter of the segmented cell. We filtered regions with 0.4 < *C* < 0.9 and regions with areas above or below 2.32 from the time-dependent mean.

### Image processing.

Tissue images captured during spatial transcriptomics tissue preparation were analyzed using a segmentation algorithm to classify each pixel into 1 of 4 categories: CLS_hi_, CLS_mid_, Other, and Adipocyte, on the basis of 3-channel pixel intensity values. Briefly, we used the Python package *skimage* to perform multi-Otsu thresholding on the 14-week red-green-blue (RGB) image tensor (51). We then extracted basic features using feature.multiscale basic features() with the following parameters: intensity = True, edges = False, texture = True, sigma min = 1, and sigma max = 16. We developed a random forest segmentation model with 50 estimators using the Python package *sklearn*. We then used the segmentation model to analyze the remaining diet conditions. Regions surrounding spatial capture spots were segmented, and the proportion of pixels in each category were computed and compared.

### Statistics.

Statistical tests included Pearson correlation, unpaired 2-tailed Student’s *t* test with Welch’s correction, and 2-tailed Wilcoxon rank-sum tests for differential gene expression, using *P* values adjusted for multiple testing using Bonferroni correction. A corrected *P* < 0.01 was considered statistically significant unless otherwise specified. Statistical tests were performed in Python using the scipy.stats library.

### Study approval.

All mouse procedures were approved by the IACUC at the University of Michigan (Animal Welfare Assurance D16-00072 [A3114-01], PRO00008583), and care was taken to minimize suffering adhering to the Institute of Laboratory Animal Research *Guide for the Care and Use of Laboratory Animals* (National Academies Press, 2011).

### Data availability.

The spatial transcriptomics and scRNA-Seq data sets generated in this study have been deposited in the Gene Expression Omnibus and can be accessed via accession number GSE198012. Source code for this investigation is available at the following link: https://github.com/CooperStansbury/spatial_transcriptomics/commit/9831b777673dbb2880df54a951f9c3d2ed15fe4e Figure data values can be found in the Supporting Data Values in supplemental materials.

## Author contributions

LAM initiated and supervised the overall project goals, designed the experiments, processed cells and tissues, and collected data on mouse metabolic function. CMS, GAD, IR, and LAM conducted formal data analysis and interpretation and created visualizations of the data. IR supervised use of computational and bioinformatics methods that were assembled and executed by CMS with input from LAM and HP. AR, IR, and LAM provided resources for the project. All authors participated in development of methodologies and writing and editing the manuscript.

## Supplementary Material

Supplemental data

Supporting data values

## Version 1 08/31/2023

In-Press Preview

## Version 2 10/09/2023

Electronic publication
