# Supplementary material for: A lipid-associated macrophage lineage rewires the spatial landscape of adipose tissue in early obesity
Source: JCI Insight. 2023 Oct 9;8(19):e171701. doi: 10.1172/jci.insight.171701 (PMC10619435; doi:10.1172/jci.insight.171701)
Supplement: Supplemental data [file jciinsight-8-171701-s179.pdf]

SUPPLEMENTARY MATERIAL

Supplementary Figures

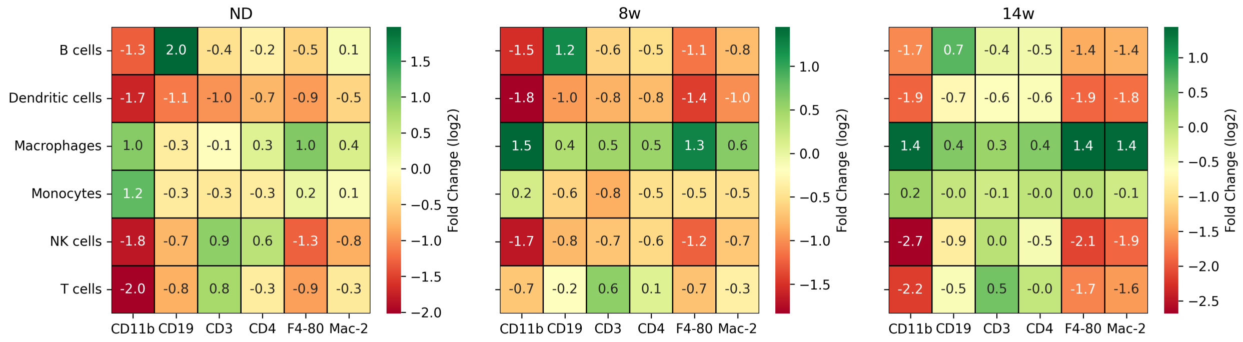

Supplementary Figure 1: **Feature barcoding summary.** Protein expression fold changes with respect to cell type annotations across all three diet conditions.

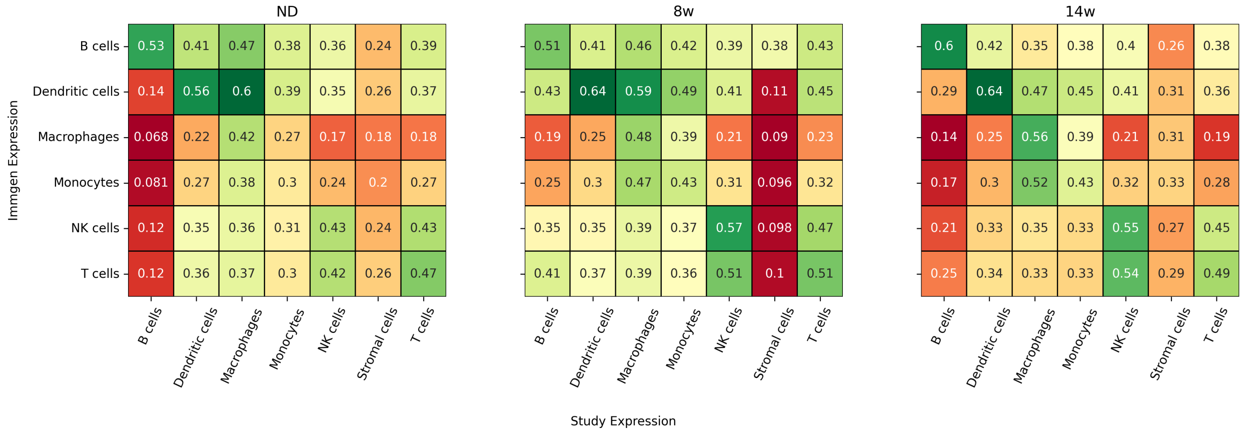

Supplementary Figure 2: **Correlation of cell type annotations with ImmGen expression profiles.** Pearson correlation of normalized expression from each cell type at each time point with ImmGen profiles (GSE122597, GSE124829, GSE75202, GSE15907, GSE75203, GSE122108, GSE37448, GSE109125, and GSE110549) for the same cell types (1).

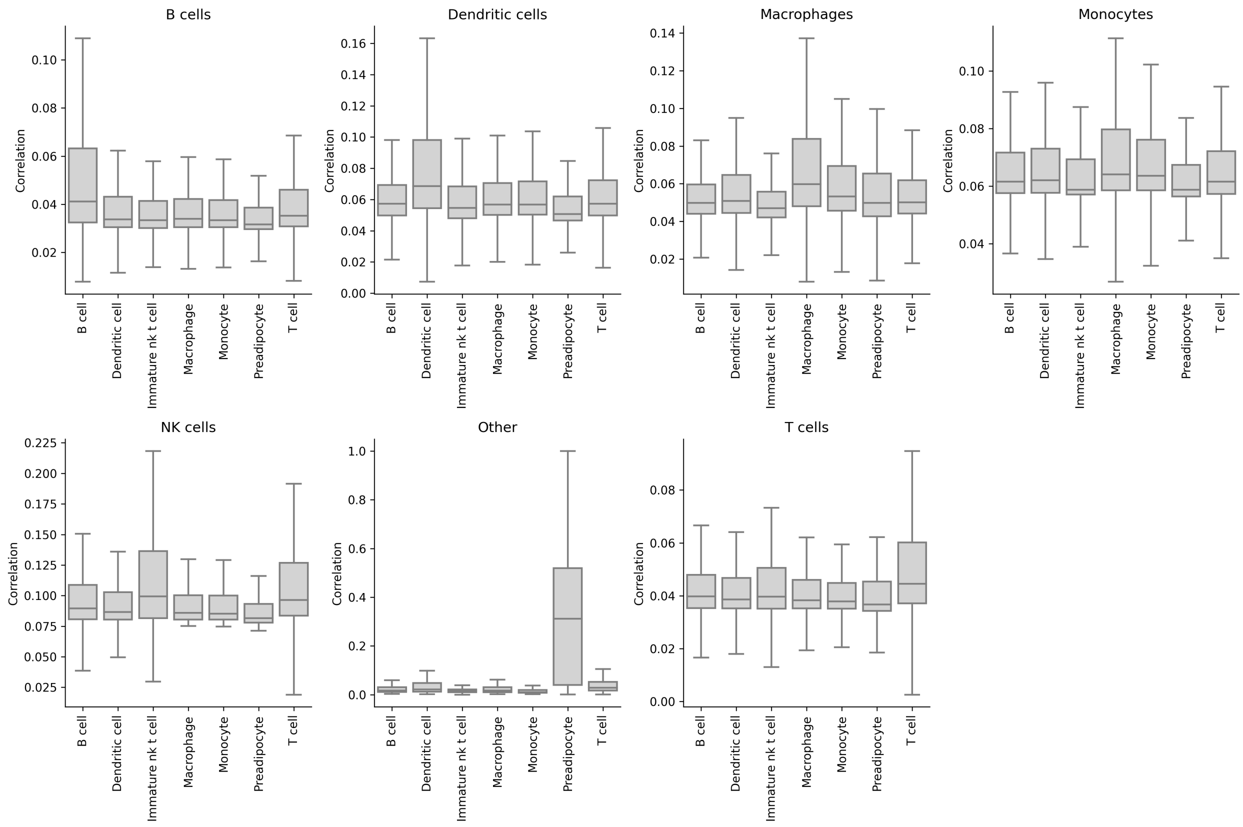

Supplementary Figure 3: **Cell type annotations align with murine eWAT scRNA-seq profiles.** Normalized expression of genes in the top 50-percentile of variance in our data was correlated with aggregated expression profiles from (2).

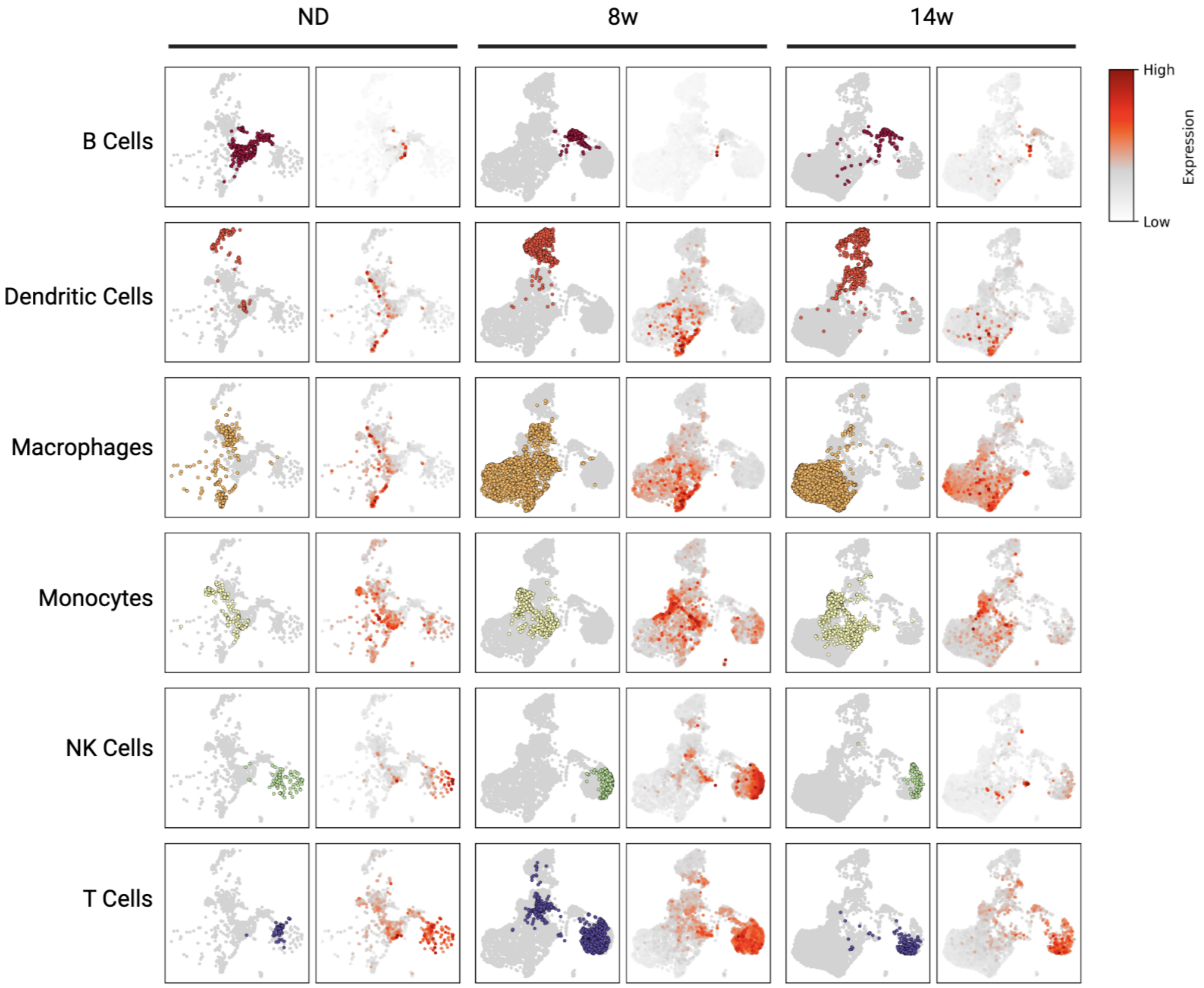

Supplementary Figure 4: **PanglaoDB marker gene expression by cell type.** Square-root mean expression of mouse marker genes from PanglaoDB (UI < 0.025) (3).

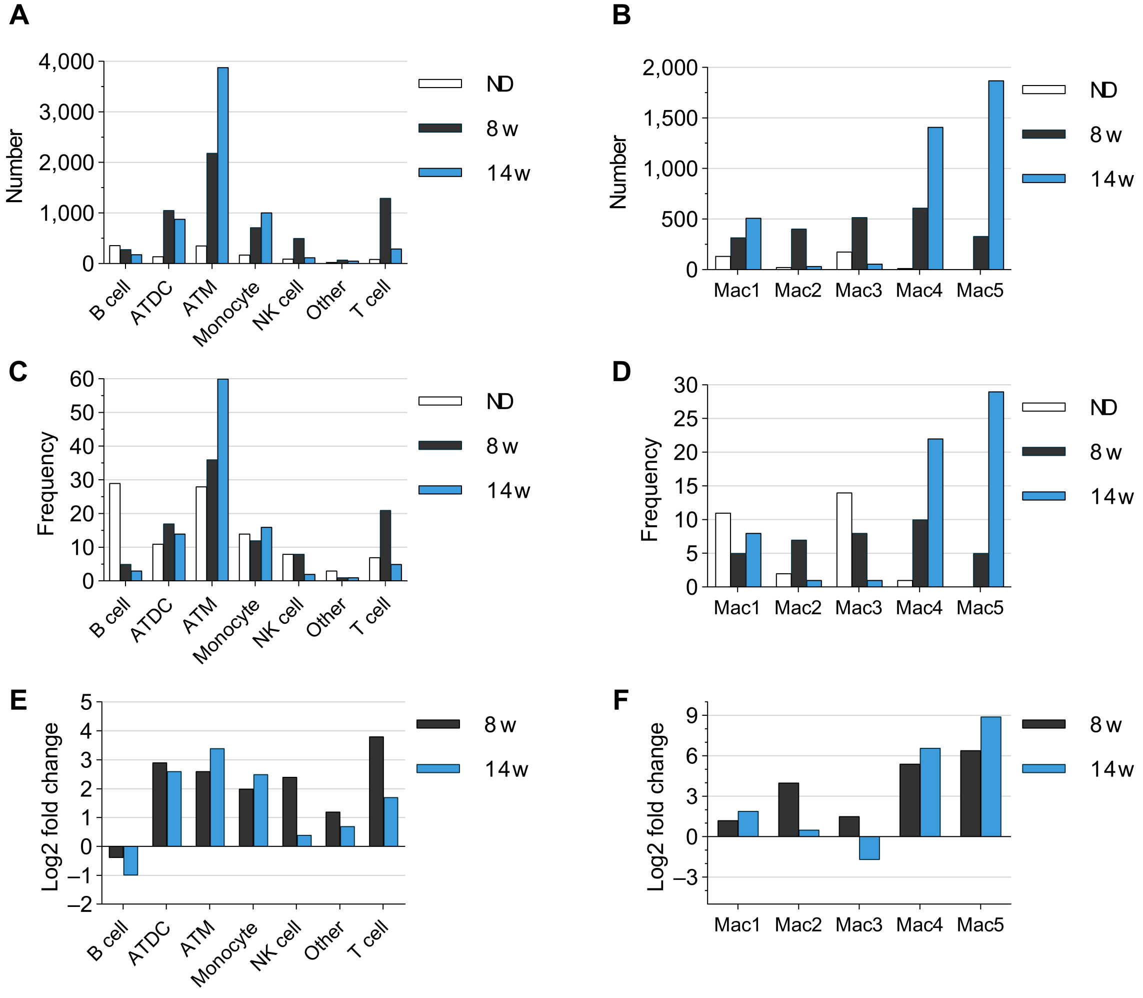

Supplementary Figure 5: **Immune cell scRNA-seq summary.** **(A)** The total number of cells of each type by diet condition. **(B)** The total number of macrophages in each subcluster by diet condition. **(C)** The frequency of each cell type by diet condition. **(D)** The frequency of each macrophage subcluster by diet condition. **(E)** The fold change (log2) of cell number over ND for all cell types. **(F)** The fold change (log2) of cell number over ND for all macrophage subtypes.

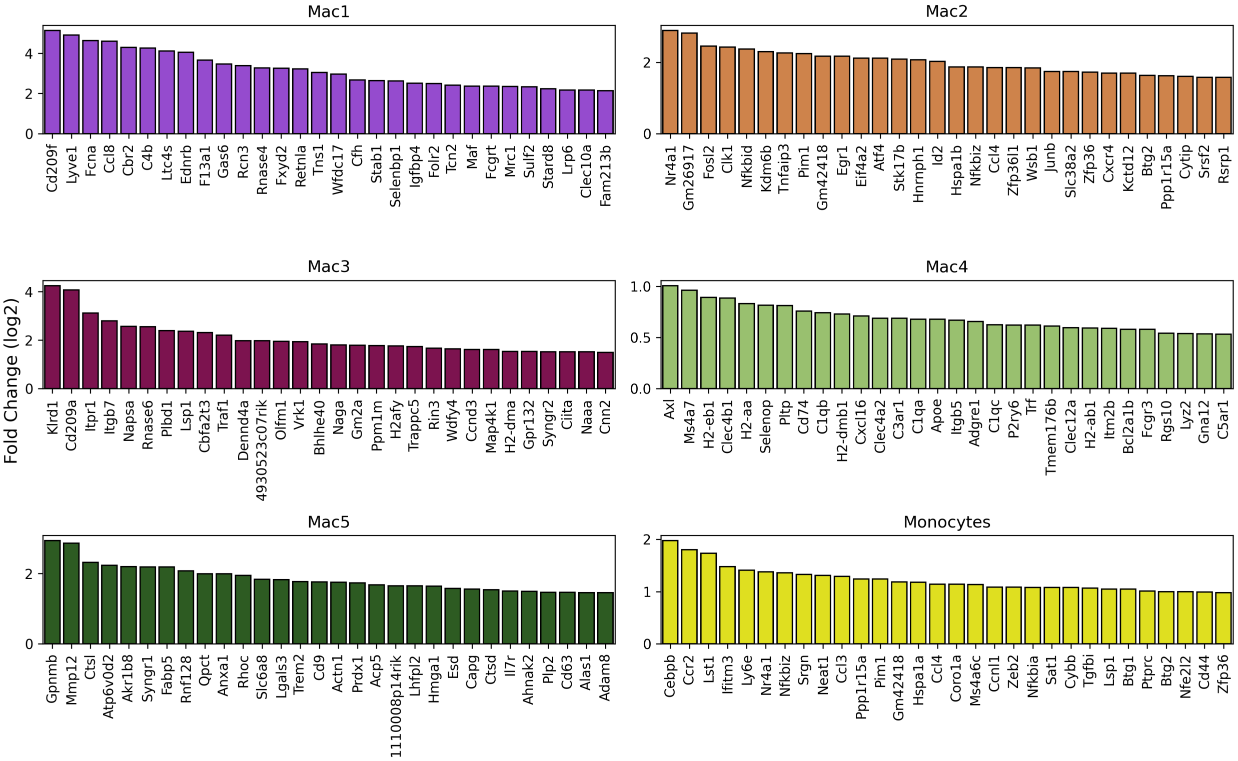

Supplementary Figure 6: **Differential expressed macrophage subtype genes.** Top differentially expressed genes from non-parametric Wilcoxon rank-sum tests adjusted using Bonferroni correction ($\alpha = 0.05$). Genes are expressed in at least 50% of the cell type, arranged by descending fold change (log2).

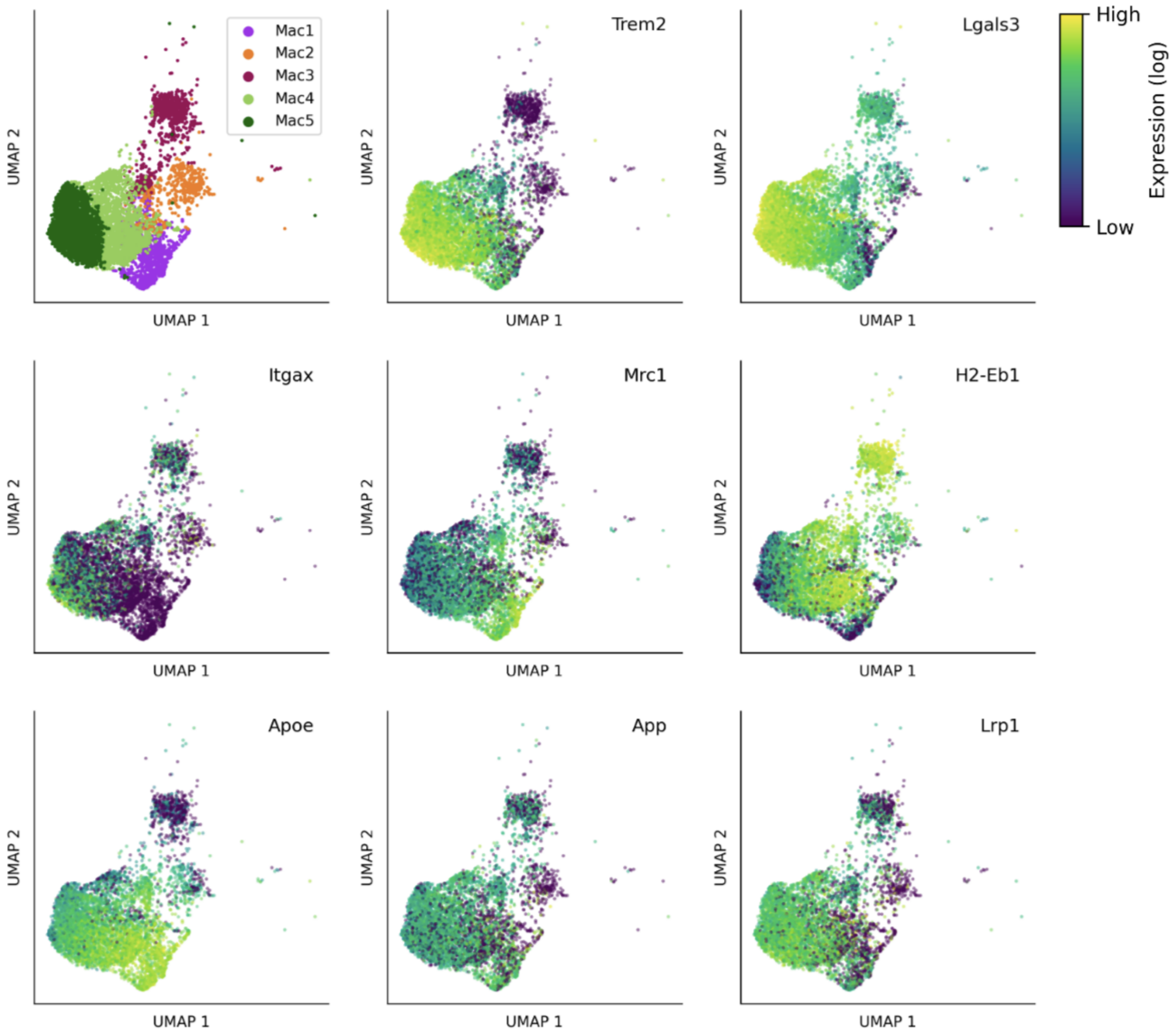

Supplementary Figure 7: **Macrophage expression of key genes.** Min-max normalized (log) expression of key genes alongside macrophage subclustering results.

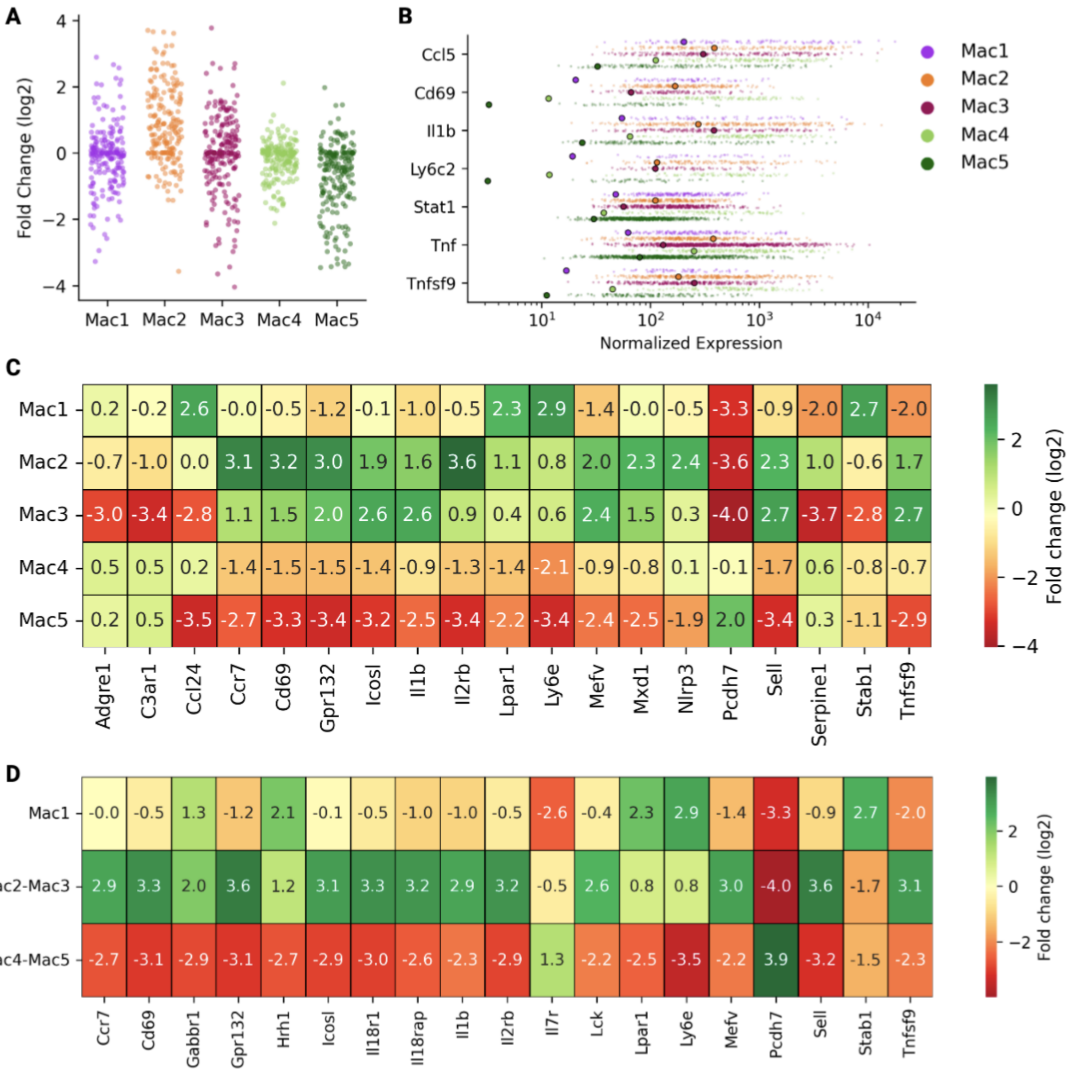

Supplementary Figure 8: **Inflammatory response-related gene expression in ATM subtypes. (A)** Distribution of log2 fold changes for 196 inflammation-related genes in the Molecular Signatures Database pathway MM3890 in ATM subtypes. Each point represents a gene. **(B)** Expression of key inflammation-related genes in macrophage subtypes. Small points represent cells and subtype means are represented by large points. **(C)** Top 15 and bottom 15 differentially expressed (Wilcoxon rank-sum test) inflammation-related genes from MM3890 across ATM subtypes. All genes are statistically significant in at least one ATM subtype using Bonferroni's correction ($\alpha = 0.05$). Genes are sorted by log2 fold change. **(D)** Top 15 and bottom 15 differentially expressed inflammation-related genes (Wilcoxon rank-sum test) for grouped ATM subtypes. All genes are statistically significant in at least one group using Bonferroni's correction ($\alpha = 0.05$). Genes are sorted by log2 fold change.

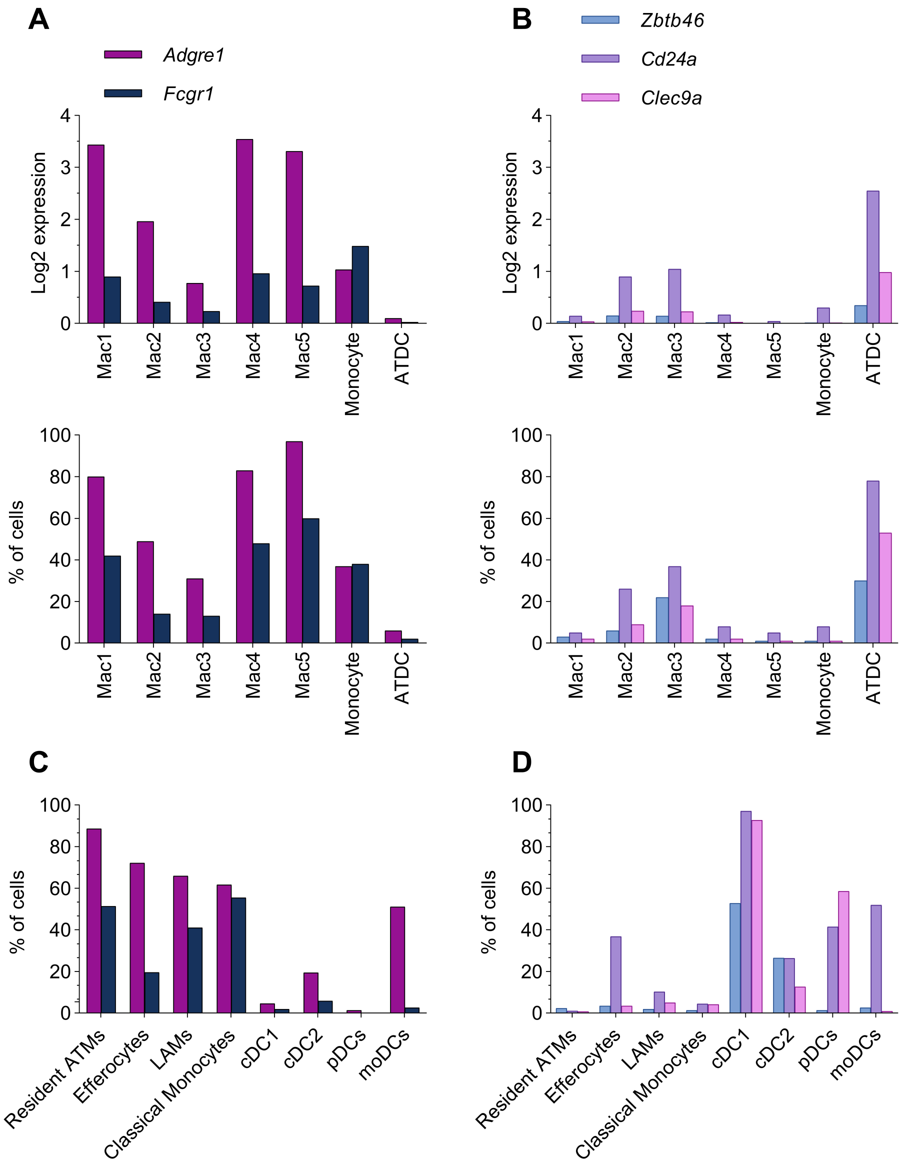

Supplementary Figure 9: **Macrophage and dendritic cell marker genes.(A)** Expression of macrophage marker genes. **(B)** Expression of dendritic cell marker genes **(C-D)** Expression of macrophage **(C)** and dendritic cell **(D)** marker genes in macrophage, monocyte, and dendritic cell subsets from (4). ATDC: adipose tissue dendritic cells.

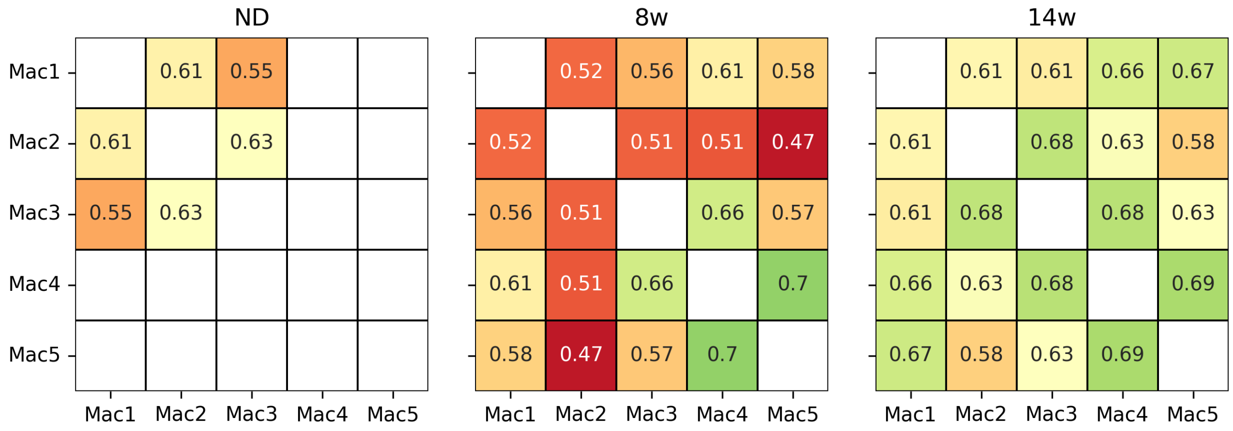

Supplementary Figure 10: **Macrophage subtype genomics correlation over time.** Pearson correlation between macrophage subtypes over PCA embedding (95% explained variance) generated from genes in at least 5% of all macrophages. Correlations are only shown when the subtype has more than 50 cells in the given diet-condition.

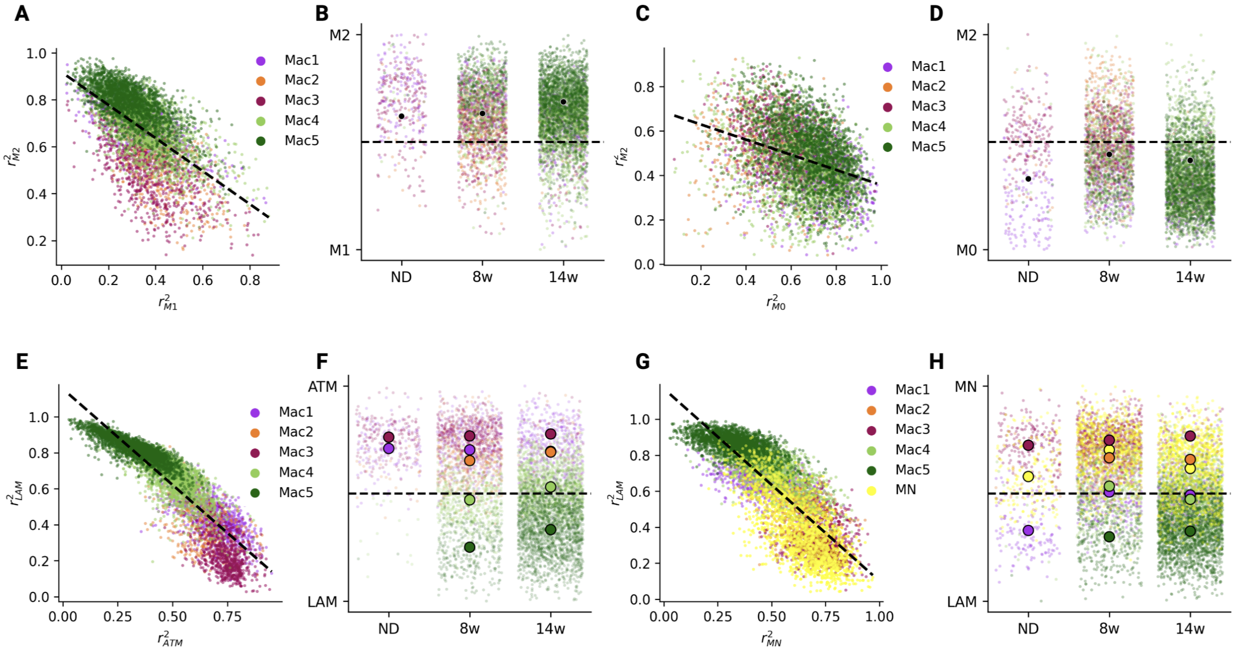

Supplementary Figure 11: **Macrophage polarization states. (A)** Correlation of macrophages to M1 and M2 macrophages from GSE117176 based on the 100 DEGs sorted by fold change (log2). **(B)** Macrophage polarization state determined by distance from the upper-left along the line of best-fit (A). **(C)** Correlation of macrophages to M2 and M0 macrophages from GSE117176 based on the 100 DEGs based on fold change (log2). Macrophage polarization state determined by distance from the upper-left along the line of best-fit (C). **(E)** Correlation of macrophages to resident macrophages (Mac1, ND) and LAM macrophages (Mac5, 14w) based on the 100 DEGs sorted by fold change (log2). **(F)** Macrophage polarization state determined by distance from the upper-left along the line of best-fit (E). **(G)** Correlation of macrophages to monocytes and LAM macrophages (Mac5, 14w) based on the 100 DEGs sorted by fold change (log2). **(H)** Macrophage polarization state determined by distance from the upper-left along the line of best-fit (G).

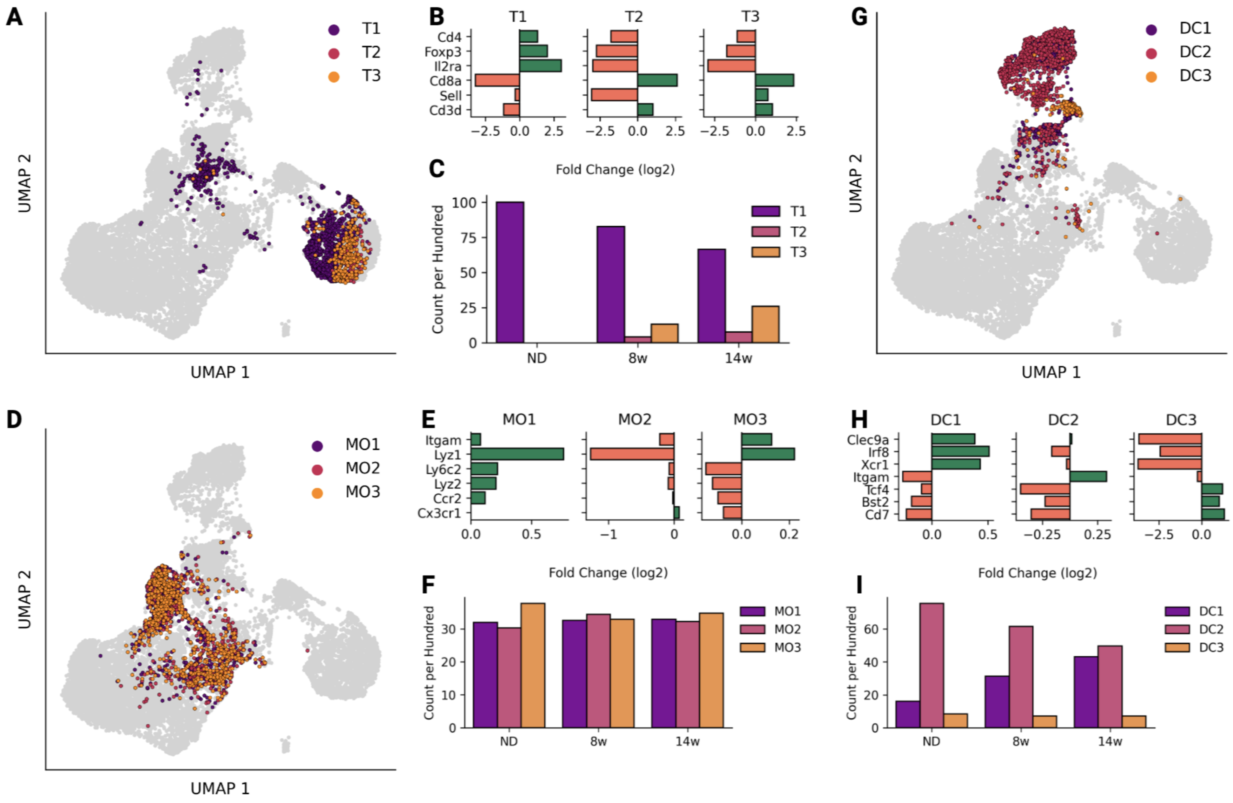

Supplementary Figure 12: **Adipose tissue T cell, monocyte, and dendritic cell subtypes in obesity by scRNA-seq.** **(A-C)** T cell subclusters included regulatory T cells (T1), conventional T cells (T2), and all other T cells (T3). **(A)** UMAP of T cell subclusters. **(B)** Differentially expressed T cell subtype markers shown as log2 fold change of each population versus all others. **(C)** T cell subtype quantity per diet condition, shown as count per hundred (proportion of parent). **(D-F)** Monocyte subclusters included inflammatory (MO1, MO3) and patrolling (MO2) subtypes. **(D)** UMAP of monocyte subclusters.**(E)** Differentially expressed monocyte subtype markers shown as log2 fold change of each population versus all others. **(F)** Monocyte subtype quantity per diet condition, shown as count per hundred (proportion of parent). **(G-I)** Dendritic cell subclusters included classical (DC1, DC2) and plasmacytoid (DC3) subtypes. **(G)** UMAP of dendritic cell subclusters. **(H)** Differentially expressed dendritic cell subtype markers shown as log2 fold change of each population versus all others. **(I)** Dendritic cell subtype quantity per diet condition, shown as count per hundred (proportion of parent). DE genes were determined using Wilcoxon rank-sum tests.

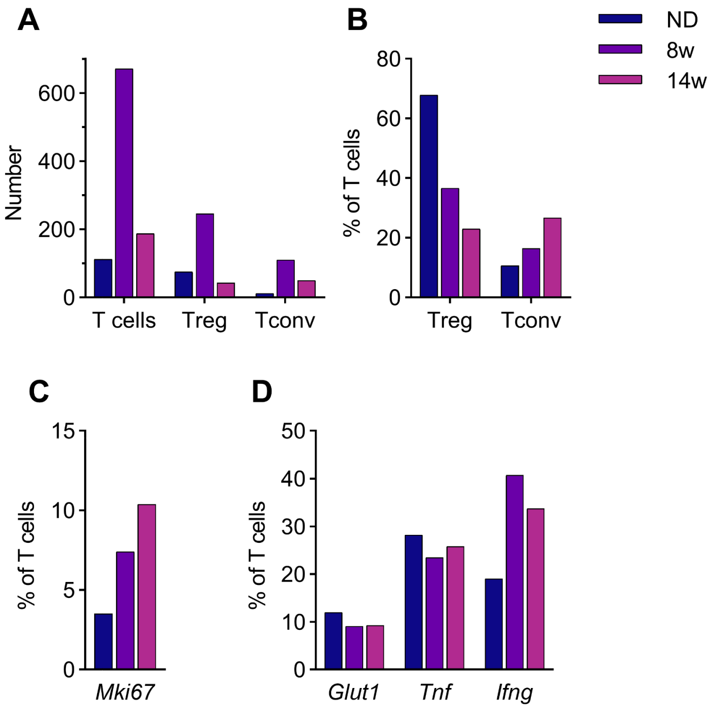

Supplementary Figure 13: **T cell activation across cohorts.** T cells were identified using classical marker gene sets in the single cell data. Regulatory T cells (Treg) were identified by expression of *Cd4, Il2ra, Foxp3, Cd27,* and *Ikzf2*, while conventional T cells (Tconv) were identified by expression of *Cd8a, Ifng, Ccl5,* and *Klrb1*. **(A)** Pooled T cell numbers for each cohort. **(B)** T cell subtype frequency. **(C)** Frequency of T cells expressing *Mki67*. **(D)** Frequency of T cells with gene expression related to activation and proinflammatory mediators.

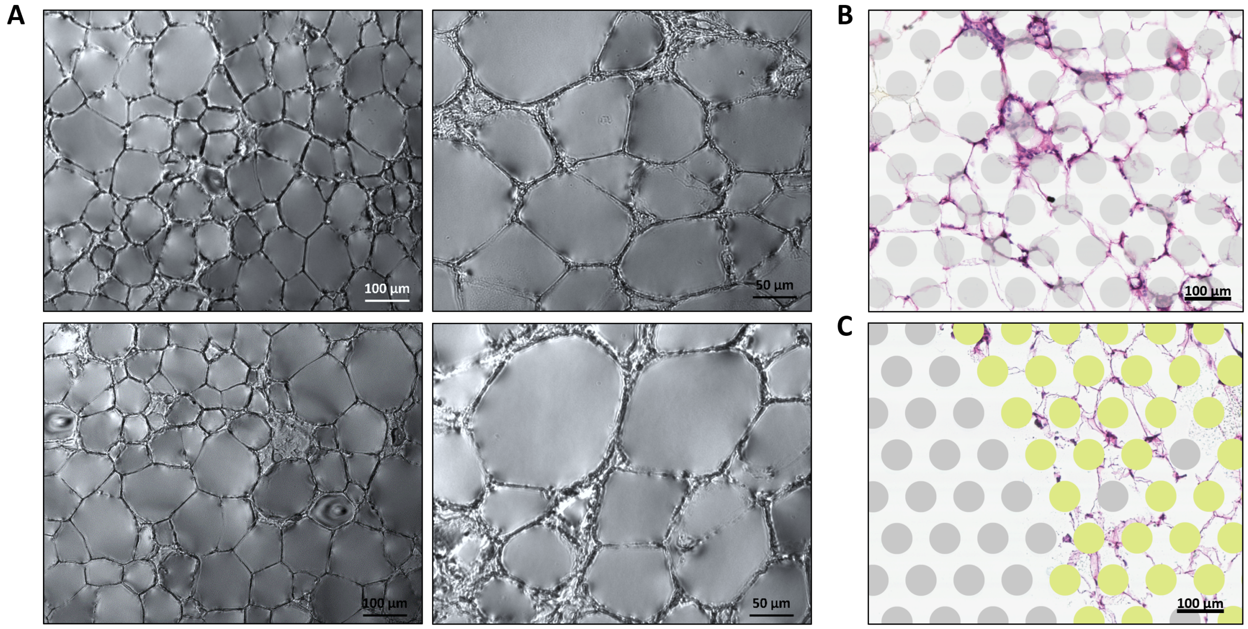

Supplementary Figure 14: **Fresh frozen adipose tissue sections after optimization.** **(A)** Ten micron sections of fresh frozen epididymal white adipose tissue from mice fed a high fat diet, shown at two different magnifications. **(B)** H&E stained section of epididymal white adipose tissue from mice fed a high fat diet for 8 weeks, used for spatial transcriptomics. Gray spots are the capture spots. **(C)** On the same slide as in (B), a field is shown spanning a tissue border to illustrate thresholding for spot quality by number of genes detected. Yellow-green spots were thresholded for expression of at least 50 genes, while gray spots have expression of fewer than 50 genes.

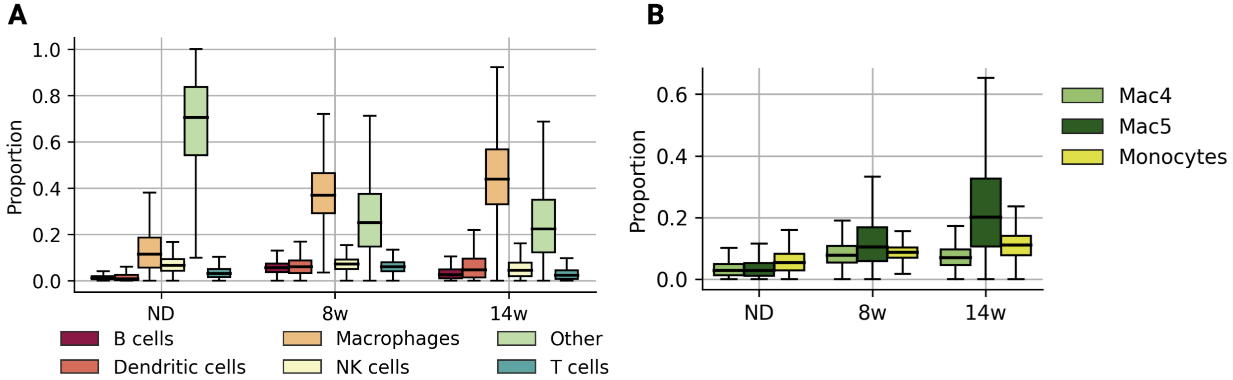

Supplementary Figure 15: **Tissue-capture spot cell-type proportions over time.** **(A)** CARD-estimated immune-cell type composition of each capture spot at each diet-condition. **(B)** CARD-estimated monocyte-LAM lineage cell-type composition at each diet condition.

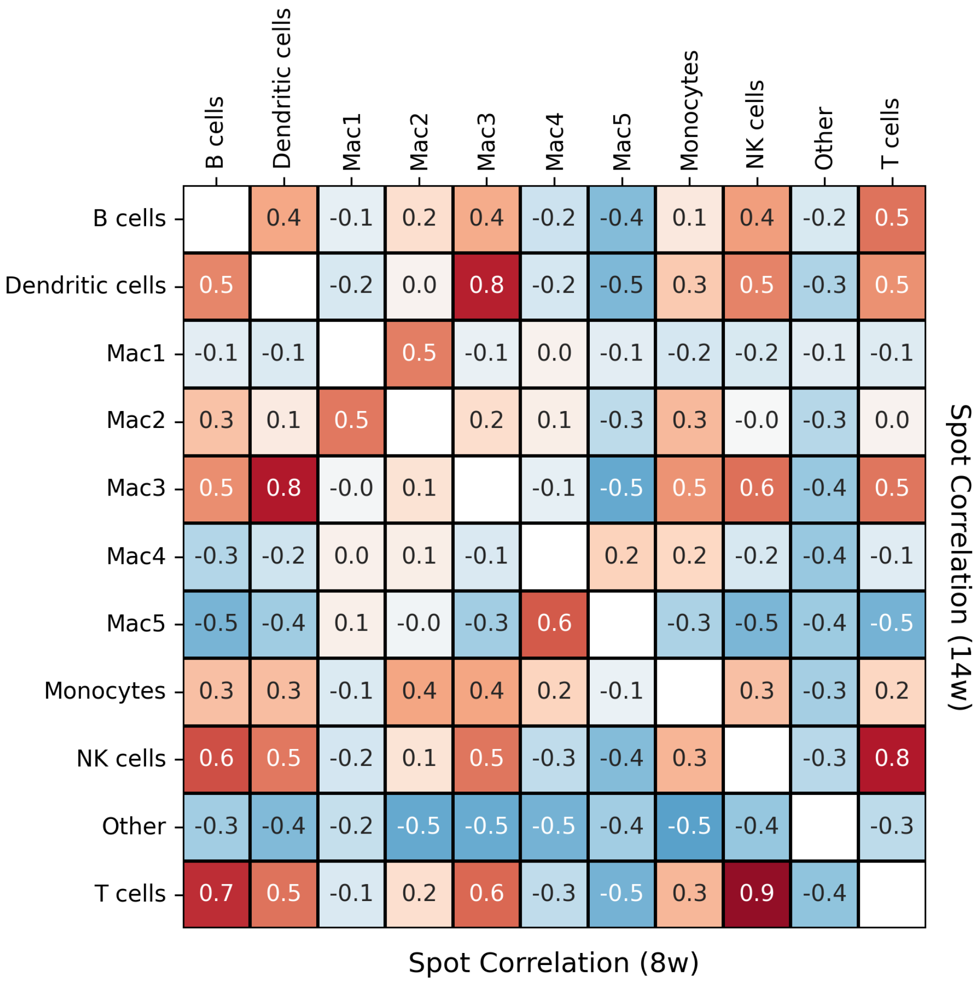

Supplementary Figure 16: **Immune-cell spot correlations.** Pearson correlation of predicted CARD proportions of immune-cell types over all tissue-capture spots at 8 weeks (below diagonal) and 14 weeks (above diagonal).

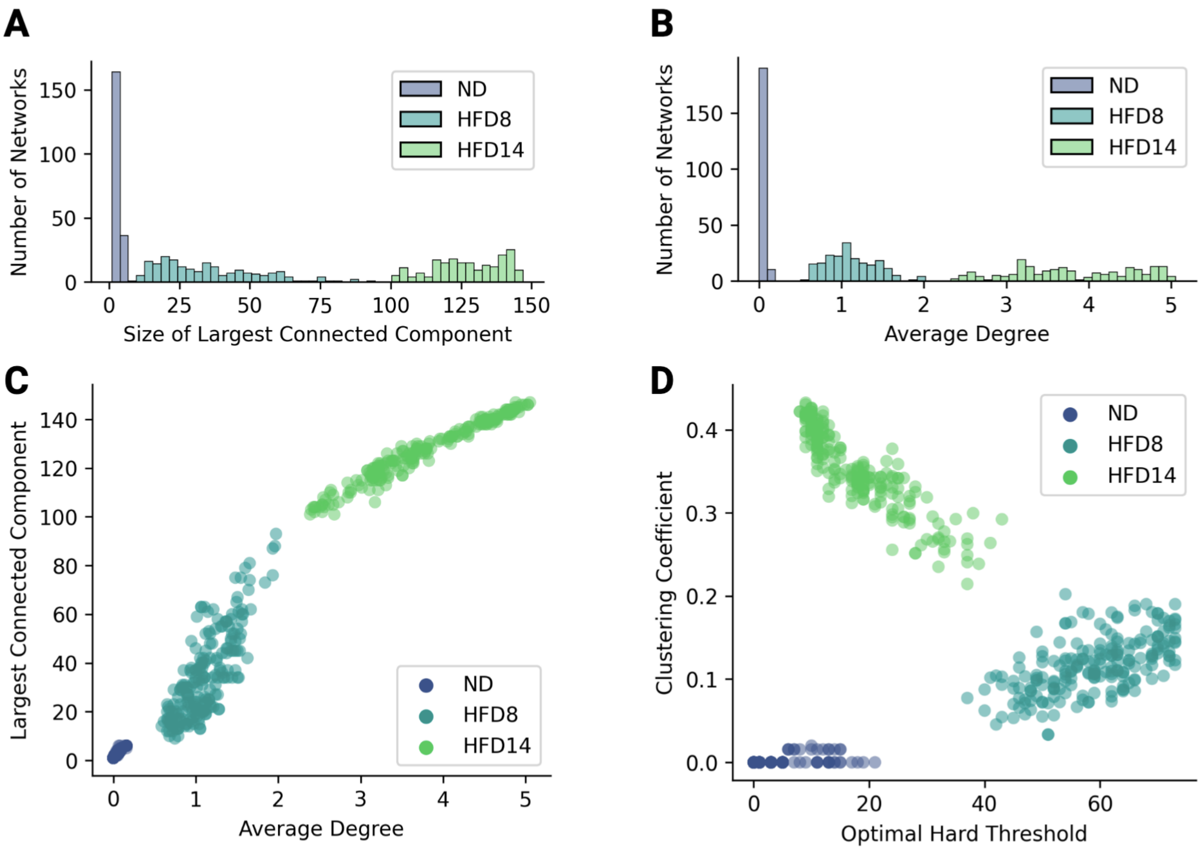

Supplementary Figure 17: **LAM networks show increased connectivity with HFD feeding. (A)** Size of largest fully connected component for LAM networks edge-thresholded at 0.015 for each diet condition. A connected component is defined by the set of nodes which can be traversed by travelling on defined edges. The largest connected component of a graph is the largest set of nodes connected by at least 1 edge. **(B)** The mean number of edges for each randomly sampled network in (A). Note that edges were defined for neighboring tissue-spots only, thus the maximum degree for a given spot is 6. We observed an increased average degree with HFD feeding, indicating more highly-localized LAM expression. **(C)** The distributions of (A) and (B) over time. **(D)** The mean clustering coefficient (5) in LAM networks compared with the optimal hard threshold (OHT) (6) of the graph's adjacency matrix **A**. The clustering coefficient is the network average of the fraction of pairs of a node's neighbors that are connected. Clustering coefficient is one for a fully connected graph but tends to zero on a random graph as the graph becomes large. The OHT is used to estimate the true rank of the graph's adjacency matrix **A**.

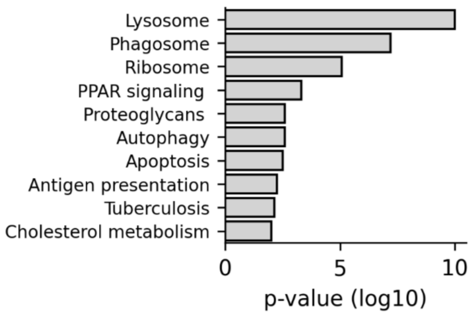

Supplementary Figure 18: **Lipid-associated macrophage network pathway analysis.** Top 10 KEGG pathways for differentially expressed genes from LAM networks at 8 weeks and 14 weeks, compared to neighboring spatial capture spots.

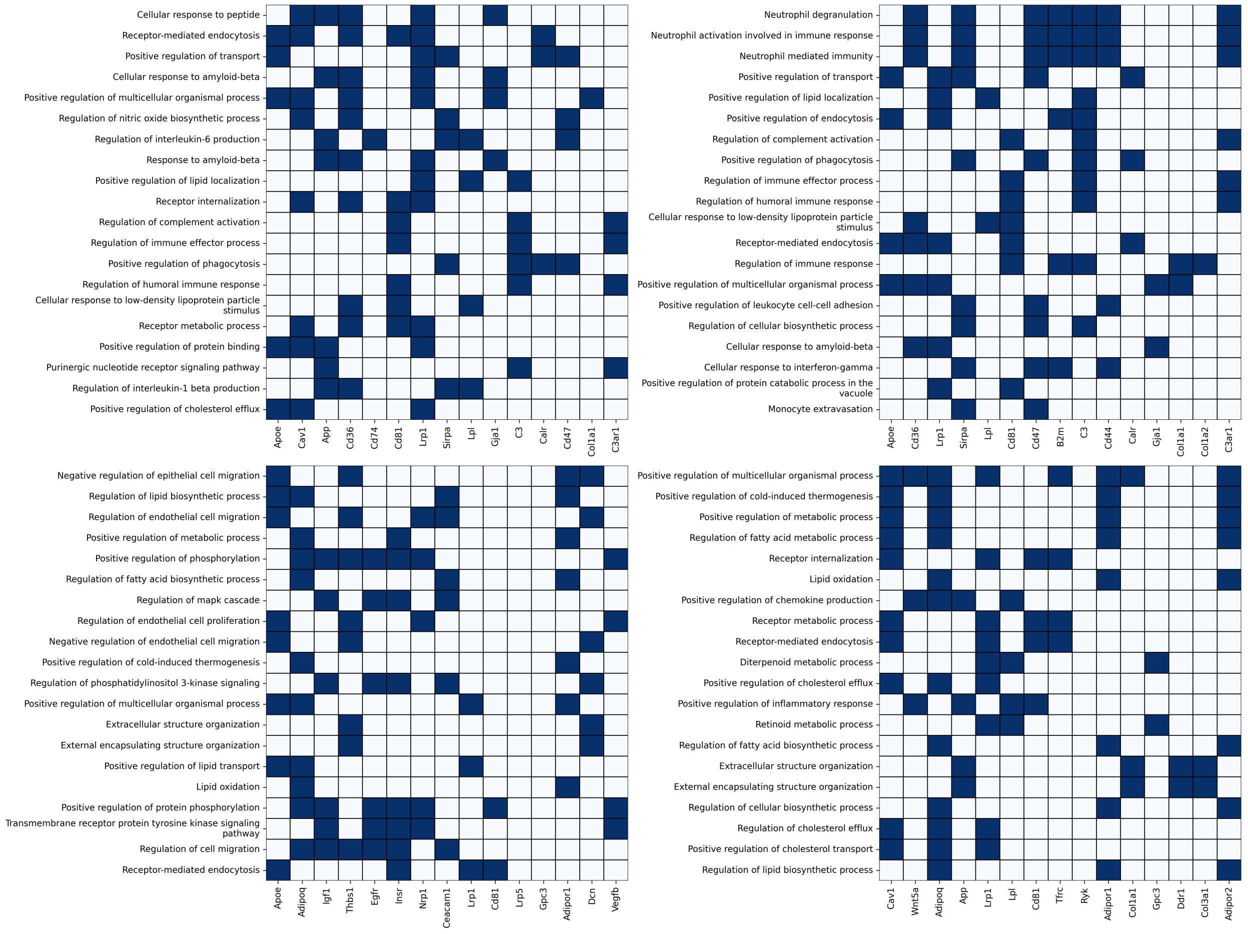

Supplementary Figure 19: **Ligand-Receptor colocalization reveals key biological processes during HFD feeding.** Enriched Gene Ontology biological processes for colocalized ligand-receptor (LR) pairs. LR pairs are divided into four groups: (upper left) increased in the first 8 weeks, (upper right) increased between 8w and 14w, (lower left) decreased in the first 8 weeks and (lower right) decreases 8w and 14.

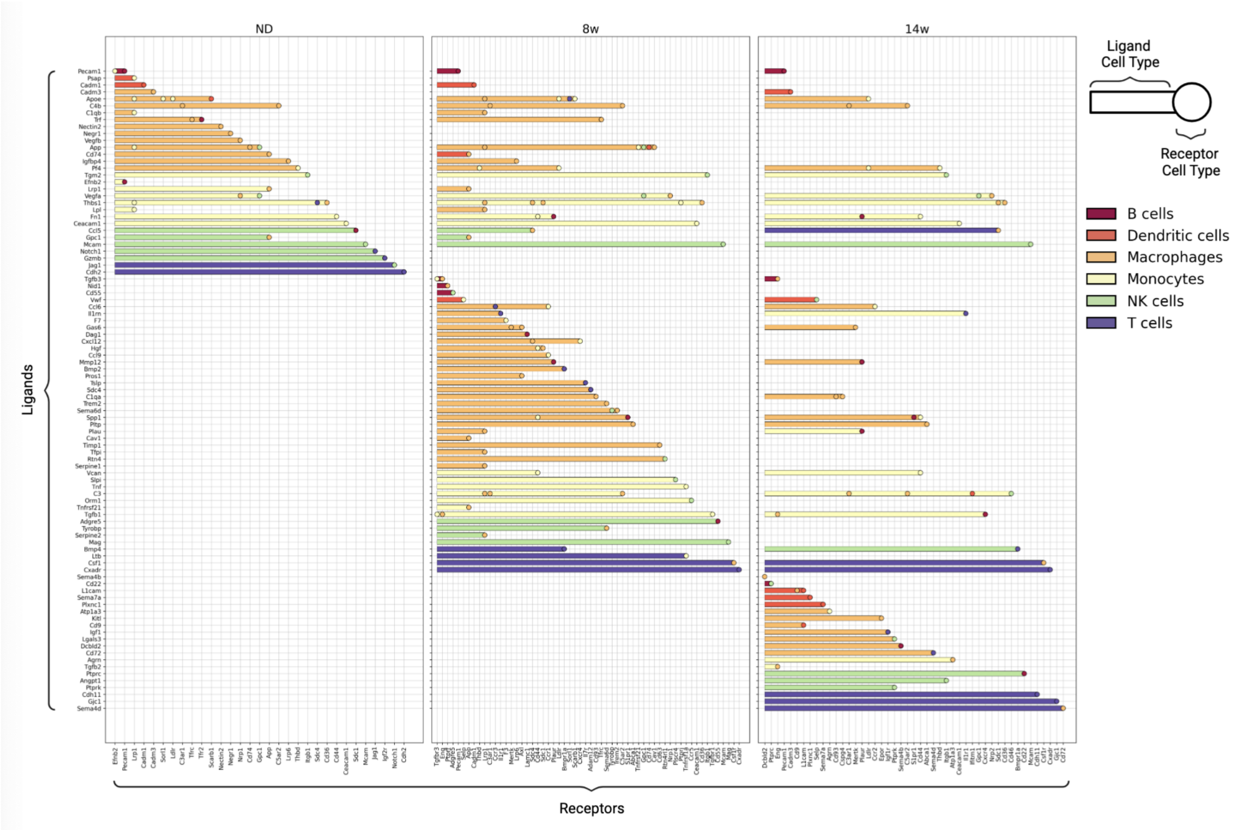

Supplementary Figure 20: **Colocalized Cell Type Specific Ligand-Receptor Expression.** Cell type specific expression of ligands (y-axis, bar color) and receptors (x-axis, point color) for each diet condition.

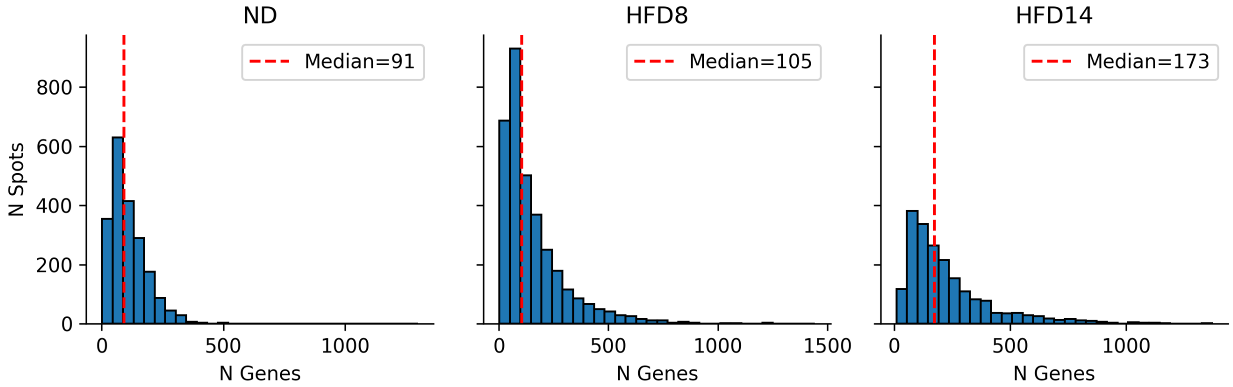

Supplementary Figure 21: **Spatial transcriptomics read depth summary.** Distribution of detected transcripts per spot in spatial transcriptomics data at each time point.

Supplementary Algorithms

Algorithm 1: Clustering and Visualization

Input: Data matrix $\mathbf{X}_{m\times n} = (\mathbf{x}_1, ..., \mathbf{x}_n) \in \mathbb{R}^{m\times n}$ where m rows are genes and n columns are cells.
Output: Cell clusters and a low dimensional projection

1: Compute the sample mean μ_n and the centered matrix $\mathbf{X}_c = \mathbf{X} - \mu_n \mathbf{1}^\top$ where $\mathbf{1}$ is a vector of ones

2: Compute the SVD of $\mathbf{X}_c = \mathbf{U}\boldsymbol{\Sigma}\mathbf{V}^\top$

3: Construct $\mathbf{P}_{n\times r} = \begin{bmatrix} v_1 & v_2 & \dots & v_r \end{bmatrix}$ where each column in $\mathbf{P}$ is a right singular vector of $\mathbf{X}_c$. Here r can be chosen using the optimal hard threshold (6) on $\mathbf{X}_c$

4: Construct a similarity matrix $\mathbf{A}_{n\times n}$ from $\mathbf{P}$ by determining the distance between each row. The choice of distance measure depends on the data type and user preference. Examples include Gaussian similarity, Euclidean distance, Manhattan distance (city block distance), Kullbeck-Liebler divergence, and correlation

5: Perform clustering: spectral or modularity clustering on $\mathbf{A}$ with k clusters. k can be chosen using domain knowledge or by testing multiple values of k and evaluating the best performance. Note: k may be $\leq r$

6: Visualization: t-SNE or UMAP to reduce the dimensions of $\mathbf{P}$ and visualize data colored according to clusters

Algorithm 2: Continuum Quantification

Input:

1. Two state matrices, $\mathbf{S}_x \in \mathbb{R}^{n_x \times m}$ and $\mathbf{S}_y \in \mathbb{R}^{n_y \times m}$ where n_x, n_y rows are the number of cells in states $\mathbf{S}_x, \mathbf{S}_y$ respectively and m columns are genes. Note that $n_x \neq n_y$, but m is assumed to be consistent between $\mathbf{S}_x$ and $\mathbf{S}_y$. The states $\mathbf{S}_x$ and $\mathbf{S}_y$ should be chosen as hypothetical poles of a continuum of biological interest.
2. Data matrix $\mathbf{D} \in \mathbb{R}^{n \times m}$ where the n rows are cells and the m columns are the genes, consistent with m above. Cells in $\mathbf{D}$ will be quantified along the continuum defined by states $\mathbf{S}_x$ and $\mathbf{S}_y$.

Output: Cell continuum values along user-defined axis for cells in $\mathbf{D}$

1: Define signatures, $\mathbf{t}_x, \mathbf{t}_y \in \mathbb{R}^m$ for states $\mathbf{S}_x$ and $\mathbf{S}_y$. For example, a function f aggregating expression of each gene over all cells:

$$\mathbf{t} = (f(\mathbf{S}))_{i=1}^{m} \,. \tag{9}$$

2: Define gene-set of interest. For example, select the top k differentially expressed genes between $\mathbf{S}_x$, and $\mathbf{S}_y$ over m, ranked by their fold change.

3: Compute the similarity between each cell and the state signatures: $\mathbf{d}_x = \text{similarity}(\mathbf{D}, \mathbf{t}_x)$ and $\mathbf{d}_y = \text{similarity}(\mathbf{D}, \mathbf{t}_y)$. The choice of similarity measure depends on the data and user preference.

4: Determine the continuum axis with respect to $\mathbf{S}_x$. For example, using ordinary least-squares (OLS), structure the following minimization problem:

$$\min_{\mathbf{w}} ||\mathbf{X}\mathbf{w} - \mathbf{d}_y||_2^2, \tag{10}$$

where $\mathbf{X}_{n\times 2} = (\mathbf{d}_x, \mathbf{1}) \in \mathbb{R}^{n\times 2}$ and $\mathbf{1}$ is a column vector of ones. The solution to Equation 10 is:

$$\mathbf{w} = \left(\mathbf{X}^\top \mathbf{X}\right)^{-1} \mathbf{X}^\top \mathbf{d}_y, \tag{11}$$

where $\mathbf{w}$ is a vector containing the slope w_0 and the intercept w_1 of the line of best fit for the data.

5: Compute the position along the continuum axis for each cell. Let $\bar{\mathbf{d}}_y$ be the predicted similarity values obtained from the OLS solution. We obtain a vector of positions along the continuum, $\bar{\mathbf{d}}_y$, using Equation 12:

$$\bar{\mathbf{d}}_y = \mathbf{X}\mathbf{w} \tag{12}$$

Let the coordinates for each cell along the continuum axis be $\mathbf{C}_{n\times 2} = (\mathbf{d}_x, \bar{\mathbf{d}}_y) \in \mathbb{R}^2$

6: Compute the distance along the continuum axis for each cell with respect to a reference point, $\mathbf{p}$. For example, the reference point may be defined as the cell with the highest similarity to either pole. Let $\mathbf{p}_{1\times 2} = (x, y) \in \mathbb{R}^2$, then the distances, $\mathbf{h}$, are defined by

$$\mathbf{h} = ||\mathbf{p} - \mathbf{C}||_2. \tag{13}$$

For convenience, we rescale distances $\mathbf{h}$ using:

$$\mathbf{h} = \frac{\mathbf{h} - \min(\mathbf{h})}{\max(\mathbf{h}) - \min(\mathbf{h})} \tag{14}$$

Supplementary Tables

Time	n Cells	Mean Area (μm)	STD Area (μm)	Mean Diameter (μm)	STD Diameter (μm)
ND	378	8,704	5,040	52.63	40.05
HFD8	663	13,004	5,853	64.33	43.16
HFD16	299	19,774	10,954	79.33	59.05

Supplementary Table 1: **Mean adipocyte size with HFD feeding.** Adipocyte area distributions measured in images from high-resolution microscopy.

Cell Type	ND	8w	14w
B cell	362	282	183
Dendritic cell	143	1,058	882
Mac1	136	317	510
Mac2	25	406	36
Mac3	179	519	57
Mac4	15	613	1,411
Mac5	4	333	1,870
Monocytes	175	714	1,009
NK cell	96	505	125
Other	33	78	54
T cell	93	1,298	299

Supplementary Table 2: **Number of cells by type at each diet condition.**

Time	Cell Type	Protein	p-value	Fold Change (log2)
ND	Macrophages	CD11b	< 0.0001	0.963
ND	Macrophages	F4-80	< 0.0001	0.996
ND	Monocytes	CD11b	< 0.0001	1.200
ND	B cells	CD19	< 0.0001	1.977
ND	NK cells	CD4	< 0.0001	0.618
ND	NK cells	CD3	< 0.0001	0.928
8w	Macrophages	CD4	< 0.0001	0.520
8w	Macrophages	CD11b	< 0.0001	1.452
8w	Macrophages	F4-80	< 0.0001	1.252
8w	Macrophages	Mac-2	< 0.0001	0.619
8w	T cells	CD3	< 0.0001	0.588
8w	B cells	CD19	< 0.0001	1.220
14w	Macrophages	CD11b	< 0.0001	1.410
14w	Macrophages	F4-80	< 0.0001	1.441
14w	Macrophages	Mac-2	< 0.0001	1.413
14w	T cells	CD3	< 0.0001	0.526
14w	B cells	CD19	< 0.0001	0.699

Supplementary Table 3: **Protein Validation of Predicted Cell Types.** Results of Wilcoxon rank-sum tests for differential protein expression for each cell type against all other cells at each time point. We adjusted α using Bonferroni's correction and required that the fold change (log2) was greater than 0.5. Using this conservative criteria we show that our cell type annotations are highly aligned with protein expression.

Cell Type 1	Cell Type 2	Number Up	Number Down
Mac1	Mac2	374	141
Mac1	Mac3	375	339
Mac1	Mac4	184	141
Mac1	Mac5	217	352
Mac1	Monocytes	394	119
Mac2	Mac3	89	234
Mac2	Mac4	170	413
Mac2	Mac5	208	932
Mac2	Monocytes	136	68
Mac3	Mac4	306	356
Mac3	Mac5	485	822
Mac3	Monocytes	145	59
Mac4	Mac5	46	52
Mac4	Monocytes	210	77
Mac5	Monocytes	546	288

Supplementary Table 4: **Number of differentially expressed genes between macrophage subtypes.** Results of pairwise differential expression analysis on genes expressed in at least 10% of the macrophage/-monocyte cell population between macrophage subtypes and monocytes. DEGs were identified using a non-parametric Wilcoxon rank sum adjusted using Bonferroni correction ($\alpha = 0.01$). We count the number of genes with fold change (log2) greater than 1 (up) and less than 1 (down).

Diet	Cell Type	μ(MN)	σ(MN)	μ(rATM)	σ(rATM)	μ(LAM)	σ(LAM)
ND	Mac5	0.396	0.112	0.160	0.083	0.981	0.025
ND	Mac4	0.587	0.115	0.219	0.182	0.719	0.123
ND	Mac3	0.716	0.087	0.246	0.099	0.299	0.077
ND	Mac2	0.525	0.133	0.402	0.145	0.373	0.155
ND	Mac1	0.244	0.099	0.783	0.129	0.286	0.132
ND	MN	0.654	0.149	0.194	0.101	0.314	0.131
8w	Mac5	0.318	0.110	0.248	0.102	0.839	0.070
8w	Mac4	0.483	0.151	0.393	0.113	0.629	0.116
8w	Mac3	0.663	0.106	0.446	0.102	0.259	0.100
8w	Mac2	0.515	0.130	0.516	0.147	0.309	0.127
8w	Mac1	0.276	0.119	0.754	0.128	0.388	0.117
8w	MN	0.667	0.156	0.374	0.068	0.262	0.150
14w	Mac5	0.325	0.116	0.307	0.121	0.842	0.069
14w	Mac4	0.424	0.128	0.447	0.100	0.694	0.092
14w	Mac3	0.679	0.127	0.543	0.141	0.443	0.097
14w	Mac2	0.502	0.142	0.597	0.161	0.454	0.119
14w	Mac1	0.307	0.121	0.767	0.107	0.521	0.106
14w	MN	0.575	0.182	0.410	0.105	0.456	0.177

Supplementary Table 5: **Macrophage Subtype Correlations.** Macrophage subtype Pearson correlations with monocyte (MN), resident ATM (rATM) and lipid-associated macropahge (LAM) gene expression signatures.

Cell Type	ND vs. 8w	ND vs. 14w	8w vs. 14w
B cell	0.000000	0.000000	0.000000
Dendritic cell	0.000000	0.000000	0.000000
Mac1	0.000000	0.028024	0.000000
Mac2	0.000000	0.000000	0.000000
Mac3	0.000000	0.000000	0.000000
Mac4	0.000000	0.000000	0.000000
Mac5	0.000000	0.000000	0.000000
Monocyte	0.000000	0.680789	0.000000
NK cell	0.632164	0.000000	0.000000
Other	0.000000	0.000000	0.000000
T cell	0.000000	0.000000	0.000000

Supplementary Table 6: **T-tests of edge weight distributions between diet-conditions.** Results for Welch's t-tests between the edge distributions of networks constructed from the entire tissue-capture area. Edge weights are defined as the harmonic mean of predicted CARD proportions between neighboring tissue-capture spots. With $\alpha = 0.01$ (Bonferonis $\hat{\alpha} = 0.0003$).

References

1. Tracy SP Heng et al. “The Immunological Genome Project: networks of gene expression in immune cells”. *Nature immunology* 9.10 (2008), 1091–1094.

2. Margo P. Emont et al. “A single-cell atlas of human and mouse white adipose tissue”. *Nature* 603.7903 (2022), 926–933.

3. Oscar Franzén, Li-Ming Gan, and Johan L M Björkegren. “PanglaoDB: a web server for exploration of mouse and human single-cell RNA sequencing data” (2019).

4. Matthew A. Cottam et al. “Multiomics reveals persistence of obesity-associated immune cell phenotypes in adipose tissue during weight loss and weight regain in mice”. *Nature Communications* 13.1 (2022), 2950.

5. Duncan J. Watts and Steven H. Strogatz. “Collective dynamics of ‘small-world’ networks”. *Nature* 393.6684 (1998), 440–442.

6. Matan Gavish and David L Donoho. “The optimal hard threshold for singular values is $4/\sqrt{3}$”. *IEEE Transactions on Information Theory* 60.8 (2014), 5040–5053.
